# Supplementary material for: GPs' attitudes, beliefs and behaviours regarding exercise for chronic knee pain: a questionnaire survey
Source: BMJ Open. 2017 Jun 17;7(6):e014999. doi: 10.1136/bmjopen-2016-014999 (PMC5541518; doi:10.1136/bmjopen-2016-014999)
Supplement: Author's manuscript [file bmjopen-2016-014999.draft_revisions.pdf]

BMJ Open

GP attitudes, beliefs and behaviours regarding exercise for chronic knee pain: a questionnaire survey

Journal:	*BMJ Open*
Manuscript ID	bmjopen-2016-014999
Article Type:	Research
Date Submitted by the Author:	02-Nov-2016
Complete List of Authors:	Cottrell, Elizabeth ; Keele University, Research Institute for Primary Care & Health Sciences<br>Foster, Nadine; Keele University, Arthritis Research UK Primary Care Centre, Research Institute of Primary Care and Health Sciences<br>Porcheret, Mark; Keele University, Arthritis Research UK Primary Care Centre, Research Institute of Primary Care and Health Sciences<br>Rathod, Trishna; Keele University, Arthritis Research UK Primary Care Centre, Research Institute of Primary Care and Health Sciences<br>Roddy, Edward; Keele University, Arthritis Research UK Primary Care Centre, Research Institute of Primary Care and Health Sciences
**Primary Subject Heading**:	General practice / Family practice
Secondary Subject Heading:	Evidence based practice, Rheumatology
Keywords:	Knee < ORTHOPAEDIC & TRAUMA SURGERY, osteoarthritis, exercise, general practitioners, surveys and questionnaires, attitude

SCHOLARONE™
Manuscripts

GP attitudes, beliefs and behaviours regarding exercise for chronic knee pain: a questionnaire survey

Elizabeth Cottrell MBChB(Hons), MRCGP, Ph.D.*^, Nadine E. Foster BSc(Hons), DPhil, FCSP*, Mark Porcheret MBBS, FRCGP, Ph.D.*, Trishna Rathod MSc*, Edward Roddy DM, FRCP*

*Arthritis Research UK Primary Care Centre, Research Institute for Primary Care and Health Sciences, Keele University, UK

^Corresponding author: Arthritis Research UK Primary Care Centre, Research Institute for Primary Care and Health Sciences, Keele University, Keele, Staffordshire, ST5 5BG.

Telephone: 01782 734870. Fax: 01782 734719. Email: e.cottrell@keele.ac.uk

KEY WORDS

Chronic knee pain, osteoarthritis, exercise, general practitioners, surveys and questionnaires, attitude

Word count excluding title page, abstract, references, tables and figures: 2862

Number of tables and figures: Figures 2, Tables 4

For peer review only

ABSTRACT

Objectives:

The aim of this study was to investigate general practitioners' (GPs) attitudes, beliefs and behaviours regarding the use of exercise for patients with chronic knee pain (CKP) attributable to osteoarthritis.

Setting:

Primary care GPs in the UK.

Participants:

5000 GPs, randomly selected from Binley's database, were mailed a cross-sectional questionnaire survey.

Outcome measures:

GPs' attitudes and beliefs were investigated using attitude statements, and reported behaviours were identified using vignette-based questions. GPs were invited to report barriers experienced when initiating exercise with patients with CKP.

Results:

835 (17%) GPs responded. Overall, GPs were positive about general exercise for CKP. 729 (87%) reported using exercise, of which, 538 (74%) reported that they would use both general and local (lower limb) exercises. However, only 92 (11% of all responding) GPs reported initiating exercise in ways aligning with best-evidence recommendations. 815 (98%) GPs reported barriers in using exercise for patients with CKP, most commonly, insufficient time in consultations (n=419; 51%) and insufficient expertise (n=337; 41%).

Conclusions:

For peer review only

While GPs' attitudes and beliefs regarding exercise for CKP were generally positive, initiation of exercise was often poorly aligned with current recommendations, and barriers and uncertainties were reported. GPs' use of exercise may be improved by addressing the key barriers of time and expertise, by developing a pragmatic approach that supports GPs to initiate individualised exercise, and/or by other professionals taking on this role.

STRENGTHS AND LIMITATIONS OF THIS STUDY

- This large UK survey is the first known to directly, specifically and concurrently investigate the attitudes, beliefs and behaviours of GPs regarding exercise for patients with CKP. Exercise initiation did not concur with best-evidence recommendations and GPs reported uncertainties and barriers in relation to using exercise.
- The use of a pre-tested vignette to investigate this topic ensured a consistent patient scenario to all GPs and minimised the confounders inherent in observational research using real patients.
- Limitations of this study include the likely over-estimation of exercise use among GPs given the low response rate (response bias), the self-report nature of the questionnaire (social desirability bias) and the relatively uncomplicated vignette case. However, as GPs should be using exercise for all patients with CKP, the results of this survey are valuable for indicating an apparent evidence-practice gap in the way in which GPs employ exercise with this patient group.

ABBREVIATIONS

CI = confidence interval; CKP = chronic knee pain; EULAR = European League Against Rheumatism; GP = general practitioners; HCP = healthcare professional; MDS = minimum data set; NICE = National Institute for Health and Care Excellence; OA = osteoarthritis; OARSI = Osteoarthritis Research Society International; OR = odds ratio; UK = United Kingdom

For peer review only

INTRODUCTION

General practitioners (GPs) are the most frequently accessed source of formal medical advice and treatment for patients in the UK with chronic knee pain (CKP).[1-3] CKP is defined in this study as being synonymous with clinical knee osteoarthritis (OA),[4] that is; mechanical knee pain, with or without loss of function, and with or without radiographic changes consistent with OA, that has lasted for at least three months in people aged 45 years and older,[5] and for which an alternative diagnosis is unlikely.[4] Globally, OA is among the leading causes of years lived with disability,[6] thus it is unsurprising that CKP is a common presentation to GPs.[7,8] Exercise, comprising of both local (lower limb focused) and general (aerobic) exercise, is recommended as core treatment for CKP,[4,9] its provision is one of the eight UK OA quality standards[10] and international OA experts recognise provision of information about regular physical activity and individualised exercise to patients as essential.[11] Empirical research evidence now unequivocally demonstrates that general aerobic, local strengthening and flexibility exercises improve pain and function in patients with CKP.[12] In line with wider self-management strategies, best practice with regards to integrating exercise into the management of patients with CKP involves providing verbal advice about both general and local exercise (which should be specific and individualised[4,13]) supported with written information.[4] Where GPs feel unable to provide specific and individualised advice, referral of patients to appropriate exercise specialists (e.g. physiotherapist) would be appropriate.

To maximise patient outcomes, GPs should align their management with best-evidence recommendations. As sociocognitive behavioural theories suggest an association between individuals' attitudes and beliefs and their behaviours,[14-16] concurrent investigation of attitudes, beliefs and behaviours of GPs was undertaken. A systematic review revealed a paucity of data specifically examining GPs use of exercise for patients with CKP, however attitudes regarding exercise were variable, it appeared to be underused and its implementation by GPs was unclear.[5] The aim of this cross-sectional questionnaire survey

For peer review only

was to identify the attitudes, beliefs and behaviours of UK GPs regarding the use of exercise for patients with CKP.

METHODS

A cross-sectional survey was used to investigate GPs attitudes, beliefs and behaviours regarding exercise for CKP. The survey tool had previously been developed through pre-testing by a local group of GPs and a subsequent pilot study with 172 UK GPs,[17] which was designed to investigate the likely response to the questionnaire, to finalise the survey tool and to test methods to maximise quantity and quality of responses.

In this main study, GPs were mailed an eight-page questionnaire (see Supplementary File 1), a cover letter and a postage-paid reply envelope in January 2014. Non-responders were sent a reminder postcard after two weeks and, four weeks after the initial mailing, persistent non-responders were mailed a second copy of the questionnaire with a cover letter and postage-paid reply envelope. At each stage non-responders who did not wish to complete the full questionnaire were invited to provide minimum data sets (MDS; gender, year of qualification, practice size and setting). Attitude statements associated with a five-point Likert scale explored GPs' attitudes and beliefs about exercise for CKP. These were minimally adapted from the work of Holden and colleagues who investigated this among physiotherapists[18] and older adults with CKP[19] and were derived from the MOVE consensus recommendations, designed to help healthcare professionals (HCPs) to initiate exercise in the management of a patient with lower limb OA[20]. GPs' reported clinical behaviour was investigated using multiple response questions associated with a vignette case (see Table 1). GPs reporting to use exercise were requested to indicate the type of exercise and how this was initiated. A multiple response item, with space for free-text, investigated GPs' experiences of barriers to using exercise for CKP. Completion and return of the questionnaire by the GP was interpreted as consent to participate in the study.

For peer review only

Table 1 Vignette used in the questionnaire to assess GPs' reported behaviours

**Patient:**	Mrs Jones, 58-year-old Prison Officer
**History:**	First presentation of gradually worsening bilateral knee pain (right worse than left) over 2 years<br>No history of trauma<br>Pain always present when walking and at rest, worst when climbing stairs. No night pain.<br>Managing activities of daily living. Difficulty gardening.<br>Stopped going to gym – thinks was making pain worse<br>Only treatment tried is ibuprofen once or twice when pain "really bad" – no benefit.<br>Came today finding work increasingly difficult due to the stairs<br>Usually well – no comorbidities
**Medication:**	Nil
**Examination:**	Body Mass Index 33<br>Knees – bilaterally no effusions. Joint tenderness upon palpation. Bilateral coarse crepitations.<br>Slightly reduced flexion of the right knee.<br>Hips **–** no abnormality detected

A minimum sample size of 288 responding GPs was required to estimate the use of exercise, based on a conservative estimate of 75% reporting exercise use informed by the pilot study[17] and a margin of error of <5%.[21] After increasing the minimum sample size to adjust for other planned regression analyses (exploring associations between reported exercise use and attitudes/beliefs (paper in preparation)) and anticipating a response rate of 20%,[17] 5000 UK GPs were randomly selected from Binley's database; a database containing the contact details of GPs in the UK which is updated quarterly. Binley's extracted a simple random sample of GPs from their database and removed and replaced any GPs included in the sample used for the previous pilot study. Study exclusion criteria were not being a GP and not having managed someone with CKP in the previous six months.

GPs' practice postcodes from each UK country were transformed into their corresponding Index of Multiple Deprivation rank[22-25] and split into quintiles (1=most deprived, 5=least deprived). Free-text responses underwent thematic analysis and responses to the GP attitude statements were condensed into three categories ((strongly) disagree, neither disagree nor agree, and (strongly) agree) before commencing descriptive analyses. Responses to GP attitude statements were interpreted as follows: unamity = 100%, consensus = 75-99%, majority view = 51-74%, no consensus = 0-50%[18]. To assess for

For peer review only

For peer review only - http://bmjopen.bmj.com/site/about/guidelines.xhtml

possible response bias in questionnaire respondents versus MDS responders, demographic data of each type of responders were compared using logistic regression to obtain unadjusted odds ratio with 95% confidence interval (CI; gender, practice area deprivation and practice type) and mean difference with 95% CI (mean years since qualification and mean number of GPs in respondents practice). An *a posteriori* analysis was undertaken due to the timing of the main survey coinciding with publication of the revised version of the NICE OA guidelines on 12th February 2014 (four weeks after the baseline mailing of the survey). To establish whether the publication of these guidelines, and the publicity associated with this event had an impact on the proportion of GPs using exercise, logistic regression was used to compare the use of exercise among responses received before the date of publication of the revised guideline with those responses received after. All analyses were performed using IBM SPSS Statistics (Version 20).

RESULTS

Response

Of the 5000 GPs sent the questionnaire, 58 responders met one or more exclusion criteria and 835 returned a completed questionnaire (adjusted response 17%). A further 470 provided MDS. The characteristics of GPs responding with a MDS were similar to those responding with a full questionnaire, except they had been qualified for longer and were more likely to work in practices in the most deprived areas (Table 2). When compared to GPs with practice postcodes in the mid-deprived quintile (OR (95% CI)), those in the most deprived (0.72 (0.60,0.87)) and second most deprived (0.76 (0.62,0.92)) were significantly less likely to respond in any way (completed questionnaire or MDS), although the absolute difference in the proportions responding were small.

For peer review only

Table 2 Demographic details of questionnaire respondents versus those providing minimum data sets

Variable	Category	Response type: MDS (n=470)	Response type: Completed questionnaire (n=835)	OR/mean difference (95% CI)
**Gender**	Male	247 (53%)	401 (49%)	OR 1.00
	Female	219 (47%)	417 (51%)	OR 1.17 (0.93,1.47)
**Practice area deprivation**	Most deprived	121 (26%)	181 (22%)	**OR 0.63 (0.45,0.89)**
	Second most deprived	106 (23%)	156 (19%)	**OR 0.62 (0.44,0.88)**
	Mid-deprived	85 (18%)	202 (24%)	OR 1.00
	Second least deprived	84 (18%)	160 (19%)	OR 0.80 (0.56,1.16)
	Least deprived	73 (16%)	135 (16%)	OR 0.78 (0.53,1.14)
**Practice type**	Urban	254 (56%)	449 (54%)	OR 1.00
	Semi-rural	155 (34%)	275 (33%)	OR 1.00 (0.78,1.29)
	Rural	43 (10%)	103 (13%)	OR 1.36 (0.92,2.00)
**Mean (SD) years since qualification**		21.64 (10.03)	18.40 (10.33)	Mean difference = -**3.24 (-2.06,-4.42)**
**Mean (SD) number of GPs in respondent's practice**		6.44 (3.67)	6.44 (3.20)	Mean difference = <0.01 (-0.38,0.39)
**Information only requested in questionnaire**				
**Type of GP**	GP partner	---	656 (79%)	---
	Salaried GP	---	151 (18%)	
	Locum GP	---	20 (2%)	
	Other	---	5 (1%)	
**GP with special interest in musculoskeletal conditions**		---	50 (6%)	---
**Received postgraduate education about CKP**		---	319 (39%)	---
**Personal experience of CKP**		----	166 (20%)	---

Maximum missing data for any cell was 6%. CI = confidence interval; CKP = chronic knee pain, GP = general practitioner; MDS = minimum data set; OR = odds ratio; SD = standard deviation

Attitudes of GPs regarding exercise for CKP

Table 3 summarises responses to GP exercise attitude statements, none were unanimous. Generally, GPs were more positive about general exercise than local exercise, particularly with regards to safety and efficacy. However, more GPs agreed that increasing the strength of the muscles around the knee stops the knee problem getting worse compared with those who agreed that increasing overall physical activity would do the same. No consensus was reached regarding the statement '*exercise works just as well for everybody, regardless of the amount of pain they have*', however the greatest proportion of respondents disagreed. GPs recognised the need to tailor exercises to individual patients, acknowledged the

For peer review only

importance of adherence with exercise but placed responsibility for adherence on the patient.

For peer review only

Table 3 Responses to GP attitude statements derived from the MOVE consensus recommendations [20]

Attitude statement	(Strongly) disagree	Neither disagree or agree	(Strongly) agree
**Items relating to the benefits of exercise (number of respondents)**			
GPs should prescribe quadriceps strengthening exercises to every patient with CKP (n=822)	8%	22%	69%
GPs should prescribe general exercise, for example, walking or swimming, for every patient with CKP (n=824)	3%	8%	89%
Knee problems are improved by quadriceps strengthening exercises (n=824)	<1%	11%	88%
Knee problems are improved by general exercise, for example, walking or swimming (n=824)	1%	7%	93%
Quadriceps strengthening exercises for the knee are safe for everybody to do (n=821)	15%	30%	56%
General exercise, for example, walking or swimming, is safe for everybody to do (n=820)	13%	16%	71%
Exercise is effective for patients if an x-ray shows severe knee osteoarthritis (n=822)	16%	32%	52%
Exercise works just as well for everybody, regardless of the amount of pain they have (n=823)	49%	29%	22%
Increasing the strength of the muscles around the knee stops the knee problem getting worse (n=824)	16%	29%	55%
Increasing the overall activity levels stops the knee problem getting worse (n=822)	19%	38%	43%
**Items relating to the delivery of, and adherence to, exercise (number of respondents)**			
Exercise for CKP is most beneficial when it is tailored to meet individual patient needs (n=823)	1%	9%	90%
A standard set of exercises is sufficient for every patient with chronic knee problems (n=821)	51%	36%	13%
GPs should educate CKP patients about how to change their lifestyle for the better (n=823)	1%	6%	93%
It is important that people with CKP increase their overall activity levels (n=824)	1%	10%	89%
How well a patient complies with their exercise programme determines how effective it will be (n=825)	3%	11%	86%
GPs should follow-up patients to monitor extent of continuation of exercises (n=823)	30%	37%	34%
It is the patient's own responsibility to continue doing their exercise programme (n=826)	1%	6%	93%

Consensus categorised according to: unamity = 100%, consensus = 75-99%, majority view = 51-74%, no consensus = 0-50% (18,26). CKP = chronic knee pain; GP = general practitioner. Maximum missing data for any item was 2%.

For peer review only

For peer review only - http://bmjopen.bmj.com/site/about/guidelines.xhtml

GPs' reported use of exercise for CKP

Of the 835 respondents, 729 (87%) reported using exercise of some type for the vignette case. Figure 1 summarises the types of exercise and initiation methods that GPs reported that they would use. Among GPs reporting to suggest general exercise (n=347), the most common recommendations were swimming (49%), walking (41%) and cycling (34%). Only 17 (5%) GPs explicitly stated that general exercise should be tailored to patient's abilities and/or interests. Among GPs reporting to use exercise, 413 (57%) stated they would achieve this by referring the patient to a physiotherapist. Table 4 cross-tabulates the exercise initiation strategies GPs reported to use for both general and local exercise and shows the most common combinations of approaches were *suggesting* general exercise and *demonstrating* local exercise, and giving the patient a leaflet about both exercise types. Thirty-two (6%) GPs reporting to use both exercise types stated they would achieve this solely by referring the patient to another HCP. Ninety-two GPs (11% of all respondents) reported to use strategies aligned with evidence-based recommendations;[4] they advised, or referred for, local and general exercise and provided written information for both exercise types (Table 4). The use of exercise was not significantly different among responses received after the publication of the revised NICE OA guidelines (273/314, 87%) when compared with those received before (456/521, 88%; OR 0.95 (95% CI 0.62,1.44)).

[INSERT Figure 1 Flow-chart summarising the exercise types and initiation methods used by GPs' for the vignette patient with CKP]

For peer review only - http://bmjopen.bmj.com/site/about/guidelines.xhtml

Table 4 Methods used to initiate local and general exercise by GPs using both exercise types

**Methods used to include general exercises**	**Methods used to include local exercises**							
	Does not demonstrate, give leaflet nor refer	***Refers only***	***Leaflet only***	***Demonstrate only***	***Demonstrate and refers***	***Leaflet and refers***	***Demonstrate and leaflet***	***Demonstrate, leaflet and refer***
Does not suggest, give leaflet nor refer	0%	<1%	0%	<1%	0%	0%	<1%	0%
Refers only	<1%	**6%**	<1%	2%	<1%	<1%	<1%	<1%
Leaflet only	0%	1%	**11%**	2%	<1%	<1%	**7%**	1%
Suggest only	0%	5%	5%	**13%**	2%	<1%	3%	<1%
Suggest and refer	<1%	1%	<1%	2%	2%	0%	<1%	0%
Leaflet and refer	0%	<1%	1%	1%	<1%	3%	2%	2%
Suggest and leaflet	<1%	<1%	3%	2%	<1%	<1%	**8%**	<1%
Suggest, leaflet and refer	0%	<1%	1%	<1%	<1%	<1%	1%	<1%

Management strategies used by >5% GPs are emboldened. The responses in the box are those that are consistent with evidence-based recommendations (i.e. providing advice and written information about both types of exercise and/or referring if needed). **n=535**

For peer review only

Use of follow-up

Of the 729 GPs reporting to use of exercise, 494 (68%) stated that they would follow-up the vignette patient to establish ongoing engagement with regular exercise. This was most commonly achieved through opportunistic follow-up (n=303, 61%) which most GPs (n=253, 84%) suggested would occur if the vignette patient failed to improve and re-consulted.

Barriers to exercise use

Most GPs (n=815, 98%) reported having experienced barriers when using exercises for patients with CKP which included: 1) service-related, 2) GP-related and 3) (perceived) patient-related barriers (Figure 2). The most frequently reported barriers were insufficient time (n=419, 51%), insufficient expertise (n=337, 41%) and the perception that patients prefer other management options (n=291, 36%).

[INSERT Figure 2 Barriers to using exercise reported by GPs]

DISCUSSION

Summary

While most GPs agreed that they should *'prescribe'* local and general exercise to all patients with CKP, believed that CKP is improved by local and general exercise, and reported that they would use exercise in the management of the vignette patient, only a tenth of responding GPs reported initiating exercise in a way that matches best-evidence recommendations.[4] This evidence-practice gap is perhaps unsurprising given that most GPs reported key barriers of time and expertise. A small number of GPs reported relying solely on referring the vignette patient to another HCP for both exercise types. This approach delays commencement of exercise as patients are not equipped to start exercising immediately after the consultation with the GP, relies on the receiving HCP delivering best practice and may not represent efficient use of services, if this is representative of GPs' usual approach for patients with CKP.

For peer review only

Comparison with existing literature

Variable GP attitudes regarding exercise for CKP were recognised in an earlier systematic review[5] and the uncertainty regarding exercise efficacy identified by the current study supports this. GPs' responses to the MOVE attitude statements from the current study were compared to those from physiotherapists[18] and older adults with CKP.[19] In contrast to GPs being generally more positive regarding general exercise when compared to local exercise, physiotherapists were generally more positive about the safety and efficacy of local exercise,[18] and older adults with CKP reported low levels of agreement about the safety and efficacy of both types of exercise.[19] Although GPs were more positive about the efficacy and safety of both exercise types than either physiotherapists or those with CKP, the timing of the respective surveys must be considered. In the time between the previous and current surveys, new and/or revised versions of NICE,[4] Osteoarthritis Research Society International (OARSI)[9] and European League Against Rheumatism (EULAR)[27] guideline recommendations for OA have been published; thus familiarity with best-evidence recommendations may have increased.

GPs' reported behaviour was consistent with higher estimates from other physician questionnaire studies; between 9-89% of GPs advising exercise[5,28-30] and 10-77% referring patients with CKP to physiotherapy[5,28-30]. Given that estimates of GPs' exercise use are similar internationally, for example, advice to exercise was 46-76% in the UK[31,32] and 12-59% in the USA[30,33-36], the results are likely to be relevant beyond the UK. The most common general exercise suggestions (swimming, walking and cycling) were clinically appropriate. Walking is acceptable to patients[37] and it improves function [38], whilst it is also low impact, easily accessible,[38] adaptable to patient preferences and easy to incorporate into everyday life. However, it is unclear how acceptable or realistic it is for patients with CKP to engage in cycling or swimming.[39,40] For example, having to pay (e.g. for equipment or instructors) is a recognised barrier to physical activity engagement.[13,41] Combining the findings of uncertain appropriateness of (some) suggested modes of exercise

For peer review only

For peer review only - http://bmjopen.bmj.com/site/about/guidelines.xhtml

with only 5% of GPs using general exercise explicitly stating that their advice should be tailored to their patients' interests and abilities, suggests a need for greater focus on individualising exercise. As many GPs reported having insufficient time and expertise to use exercise, referral to other HCPs may be appropriate. However physiotherapists report similar uncertainties about the safety and efficacy of exercise[18] and suboptimal use of both exercise types.[42] Therefore, relying on physiotherapists to deliver exercise interventions may not ensure all patients receive tailored, specific instruction on both exercise types. Finally, a third of respondents reported that they would opportunistically follow-up the vignette patient to check adherence to exercise. However, previous research examining the consultation behaviour of patients with CKP has shown that while many patients may consult again with other health problems, CKP is often not recorded again.[43] Thus the GPs' reported intentions regarding follow-up are likely to be unrealistically optimistic.

Strengths and limitations

This large UK survey is the first known to directly, specifically and concurrently investigate the attitudes, beliefs and behaviours of GPs regarding exercise for patients with CKP. Employing a vignette provided a consistent patient scenario to all GPs[44-47] and minimised the confounders inherent in observational research using real patients.[45,46] Limitations of this study include the likely over-estimation of exercise use among GPs given the low response rate (response bias), the self-report nature of the questionnaire (social desirability bias) and the relatively uncomplicated vignette case. However, as GPs should be using exercise for all patients with CKP, the results of this survey are valuable for indicating an apparent evidence-practice gap in the way in which GPs employ exercise with this patient group.

Implications for future practice

To deliver best practice for patients with CKP, strategies which target both GPs and the wider primary care team are needed. Two key areas should be addressed: i) development of

For peer review only - http://bmjopen.bmj.com/site/about/guidelines.xhtml

a pragmatic approach for GPs to initiate individualised local and general exercise and ii) identification of additional methods of initiating exercise and/or supporting patients to continue with exercise that do not solely rely on GPs. Given that theoretical patient behaviour change models[48] often involve a balance of perceived value and risks/burdens of undertaking the new behaviour, a pragmatic approach for GPs initiating exercise among patients with CKP would need to highlight the value of exercise to patients, its role relative to other interventions and practical ways to undertake specific and individualised exercise. Such a best practice approach was suggested by Khan et al[49] who recommended that GPs should encourage the use of exercise (e.g. by asking about physical activity at each consultation), consider the '5 A's' of physical activity counselling (assess, advise, agree, assist and arrange),[50] write an exercise prescription,[51-53] and refer or signpost to appropriate professionals or resources for exercise support and/or follow-up. Supplementary written leaflets or 'guidebooks' seem to be acceptable and useful[54] and can be accessed or signposted within consultations; for example, the Keele University OA guidebook[55] and the Arthritis Research UK knee OA booklet.[56] Given that CKP patients commonly have comorbidities[37,57,58] and multiple joint pain, GPs could use this opportunity to detect and manage comorbid conditions that may directly impact the use of exercise (e.g. cardiovascular disease, depression[59]), to relay the synergistic benefits of exercise for all relevant morbidities, and to make explicit that CKP should not prevent exercise for other conditions. Practical methods to help GPs provide the above information, in limited available time, need to be developed and may include personalised written care plans.[60] Given that time was the most frequently reported barrier to GPs initiating exercise, service delivery models may need to change such that exercise initiation, support and/or follow-up is primarily undertaken by other professionals such as physiotherapists,[61,62] practice nurses,[63] health trainers or local gym personnel. Direct access may enhance patients' utilisation of physiotherapists[64] and alternative, low GP burden, strategies could be explored to promote exercise use and/or follow-up. For example, technology enabled care

For peer review only - http://bmjopen.bmj.com/site/about/guidelines.xhtml

For peer review only

services[65] have shown promise when used to support exercise interventions among patients with cardiac[66] and chronic lung disease.[67]

CONCLUSIONS

Although the majority of UK GPs who responded to the questionnaire survey were positive about exercise for patients with CKP and used exercise in their clinical management, this survey identified GP uncertainties with respect to the safety and efficacy of exercise and suboptimal approaches to the initiation of exercise with patients. GPs' use of exercise may be improved by addressing the key barriers of time and expertise, by developing a pragmatic approach that supports GPs to initiate individualised exercise with patients, and/or by other professionals taking on this role.

ACKNOWLEDGEMENTS

We would like to thank the staff within the Research Institute for Primary Care and Health Sciences who supported the study. Thanks also to the GPs who participated.

CONTRIBUTORSHIP STATEMENT

EC, NF, MP, TR, and ER participated in the design of the study, analysis of the results and helped to draft the manuscript. All authors read and approved the final manuscript.

COMPETING INTERESTS

The authors declare that they have no competing interests.

DATA SHARING

The datasets analysed during the current study available from the corresponding author on reasonable request.

FUNDING

For peer review only

This paper presents independent research funded by the Arthritis Research UK Centre in Primary Care grant (Grant Number 20202). NF, an NIHR Senior Investigator, is supported through an NIHR Research Professorship (NIHR-RP-011-015). EC was funded by an NIHR Academic Clinical Fellowship and subsequently by School for Primary Care Research bridging funds. The views expressed in this publication are those of the authors and not necessarily those of the NHS, the NIHR or the Department of Health.

ETHICAL APPROVAL

Ethical approval for the study was obtained from Keele University (UK) Ethical Review Panel.

REFERENCES

(1) Jordan KM, Sawyer S, Coakley P, Smith HE, Cooper C, Arden NK. The use of conventional and complementary treatments for knee osteoarthritis in the community. *Rheumatology (Oxford)* 2004;43(3):381-4.

(2) Jinks C, Ong BN, Richardson J. A mixed methods study to investigate needs assessment for knee pain and disability: population and individual perspectives. *BMC Musculoskelet Disord* 2007;8:59.

(3) Thorstensson CA, Gooberman-Hill R, Adamson J, Williams S, Dieppe P. Help-seeking behaviour among people living with chronic hip or knee pain in the community. *BMC Musculoskelet Disord* 2009;10:153.

(4) National Institute for Health and Care Excellence. Osteoarthritis: care and management in adults. 2014;CG177.

(5) Cottrell E, Roddy E, Foster NE. The attitudes, beliefs and behaviours of GPs regarding exercise for chronic knee pain: a systematic review. *BMC Fam Pract* 2010;11(4).

(6) Vos T, Barber RM, Bell B, Bertozzi-Villa A, Biryukov S, Bolliger I, et al. Global, regional, and national incidence, prevalence, and years lived with disability for 301 acute and chronic diseases and injuries in 188 countries, 1990–2013: a systematic analysis for the Global Burden of Disease Study 2013. *Lancet* 2015 2016/03;386(9995):743-800.

(7) Jordan KP, Kadam UT, Hayward R, Porcheret M, Young C, Croft P. Annual consultation prevalence of regional musculoskeletal problems in primary care: an observational study. *BMC Musculoskelet Disord* 2010;11:144.

(8) Arthritis Research UK National Primary Care Centre. Musculoskeletal matters. Consultations for selected diagnoses and regional problems. Musculskeletal Matters 2010;Bulletin 2.

For peer review only

For peer review only - http://bmjopen.bmj.com/site/about/guidelines.xhtml

(9) McAlindon TE, Bannuru RR, Sullivan MC, Arden NK, Berenbaum F, Bierma-Zeinstra SM, et al. OARSI guidelines for the non-surgical management of knee osteoarthritis. *Osteoarthritis Cartilage* 2014;22(3):363-88.

(10) National Institute for Health and Care Excellence. Osteoarthritis NICE quality standard. QS87 ed. http://www.nice.org.uk/guidance/qs87: NICE; 2015.

(11) French SD, Bennell KL, Nicolson PJ, Hodges PW, Dobson FL, Hinman RS. What do people with knee or hip osteoarthritis need to know? An international consensus list of essential statements for osteoarthritis. *Arthritis Care Res (Hoboken)* 2015 May;67(6):809-16.

(12) Uthman OA, van der Windt DA, Jordan J, Dziedzic KS, Healey EL, Peat GM, et al. Exercise for lower limb osteoarthritis: systematic review incorporating trial sequential analysis and network meta-analysis. *BMJ* 2013;347:f5555.

(13) Mazieres B, Thevenon A, Coudeyre E, Chevalier X, Revel M, Rannou F. Adherence to, and results of, physical therapy programs in patients with hip or knee osteoarthritis. Development of French clinical practice guidelines. *Joint Bone Spine* 2008;75(5):589-96.

(14) Ajzen I. The theory of planned behavior. *Organ Behav Hum Decis Process* 1991 12;50(2):179-211.

(15) Michie S, Johnston M, Abraham C, Lawton R, Parker D, Walker A, et al. Making psychological theory useful for implementing evidence based practice: a consensus approach. *Qual Saf Health Care* 2005;14(1):26-33.

(16) Godin G, Bélanger-Gravel A, Eccles M, Grimshaw J. Healthcare professionals' intentions and behaviours: a systematic review of studies based on social cognitive theories. *Implement Sci* 2008;3:36.

(17) Cottrell E, Roddy E, Rathod T, Thomas E, Porcheret M, Foster N. Maximising response from GPs to questionnaire surveys: do length or incentives make a difference? *BMC Med Res Methodol* 2015;15(1):3.

(18) Holden MA, Nicholls EE, Young J, Hay EM, Foster NE. UK-based physical therapists' attitudes and beliefs regarding exercise and knee osteoarthritis: findings from a mixed-methods study. *Arthritis Rheum* 2009;61(11):1511-21.

(19) Holden MA, Nicholls EE, Young J, Hay EM, Foster NE. Role of exercise for knee pain: what do older adults in the community think? *Arthritis Care Res (Hoboken)* 2012;64(10):1554-64.

(20) Roddy E, Zhang W, Doherty M, Arden NK, Barlow J, Birrell F, et al. Evidence-based recommendations for the role of exercise in the management of osteoarthritis of the hip or knee - the MOVE consensus. *Rheumatology (Oxford)* 2005;44(1):67-73.

(21) Zar J. Biostatistical analysis. 4th ed.: Prentice Hall; 1998.

(22) Northern Ireland Statistics & Research Agency. Northern Ireland Multiple Deprivation Measure 2010. 2010; Available at: http://www.nisra.gov.uk/deprivation/nimdm_2010.htm. Accessed January/06, 2015.

For peer review only - http://bmjopen.bmj.com/site/about/guidelines.xhtml

(23) Department for Communities and Local Government. English indices of deprivation 2010. 2011; . Accessed December/12, 2014.

(24) Welsh Government. Welsh Index of Multiple Deprivation (WIMD). 2014; Available at: http://wales.gov.uk/statistics-and-research/welsh-index-multiple-deprivation/?lang=en. Accessed January/06, 2015.

(25) The Scottish Government. Scottish Index of Multiple Deprivation. 2014; Available at: http://www.scotland.gov.uk/Topics/Statistics/SIMD. Accessed December/12, 2014.

(26) Mercer C, Jackson A, Moore A. Developing clinical guidelines for the physiotherapy management of whiplash associated disorder (WAD). *Int J Osteopath Med* 2007 2014/12;10(2):50-4.

(27) Fernandes L, Hagen KB, Bijlsma JWJ, Andreassen O, Christensen P, Conaghan PG, et al. EULAR recommendations for the non-pharmacological core management of hip and knee osteoarthritis. *Ann Rheum Dis* 2013;72(7):1125-35.

(28) Arshad A, Rashid R, Das Gupta E. A survey of the primary care management of osteoarthritis is Malaysia: a view from a rheumatologist's perspective. *Int J Rheum Dis* 2008;11(3):246-50.

(29) Richette P, Hilliquin P, Bertin P, Carni P, Berger V, Marty M. Comparison of general practitioners and rheumatologists' prescription patterns for patients wtih knee osteoarthritis. *BMC Musculoskelet Disord* 2011;12:72.

(30) Maserejian NN, Fischer MA, Trachtenberg FL, Yu J, Marceau LD, McKinlay JB, et al. Variations among primary care physicians in exercise advice, imaging, and analgesics for musculoskeletal pain: Results from a factorial experiment. *Arthritis Care Res (Hoboken)* 2014;66(1):147-56.

(31) Bedson J, Jordon K, Croft P. How do GPs use x rays to manage chronic knee pain in the elderly? A case study. *Ann Rheum Dis* 2003;62(5):450-4.

(32) Porcheret M, Jordan K, Croft P. Treatment of knee pain in older adults in primary care: development of an evidence-based model of care. *Rheumatology (Oxford)* 2007;46(4):638-48.

(33) Dexter PA. Joint exercises in elderly persons with symptomatic osteoarthritis of the hip or knee. Performance patterns, medical support patterns, and the relationship between exercising and medical care. *Arthritis Care Res* 1992;5(1):36-41.

(34) Mazzuca SA, Brandt KD, Katz BP, Dittus RS, Freund DA, Lubitz R, et al. Comparison of general internists, family physicians and rheumatologists managing patients with symptoms of osteoarthritis of the knee. *Arthritis Care Res* 1997;10(5):289-99.

(35) Glauser TA, Salinas GD, Roepke NL, Chad Williamson J, Reese A, Gutierrez G, et al. Management of mild-to-moderate osteoarthritis: a study of the primary care perspective. *Postgrad Med* 2011;123(1):126-34.

(36) Mamlin LA, Melfi CA, Parchman ML, Gutierrez B, Allen DI, Katz BP, et al. Management of osteoarthritis of the knee by primary care physicians. *Arch Fam Med* 1998;7(6):563-7.

For peer review only

For peer review only - http://bmjopen.bmj.com/site/about/guidelines.xhtml

(37) Holden MA, Nicholls EE, Young J, Hay EM, Foster NE. Exercise and physical activity in older adults with knee pain: a mixed methods study. *Rheumatology (Oxford)* 2015;54(3):413-23.

(38) O'Connor SR, Tully MA, Ryan B, Bleakley CM, Baxter GD, Bradley JM, et al. Walking exercise for chronic musculoskeletal pain: systematic eeview and meta-analysis. *Arch Phys Med Rehabil* 2015;96(4):724-34.e3.

(39) Hendry M, Williams NH, Markland D, Wilkinson C, Maddison P. Why should we exercise when our knees hurt? A qualitative study of primary care patients with osteoarthritis of the knee. *Fam Pract* 2006;23(5):558-67.

(40) Bossen D, Veenhof C, Dekker J, de Bakker D. The usability and preliminary effectiveness of a web-based physical activity intervention in patients with knee and/or hip osteoarthritis. *BMC Med Inform Decis Mak* 2013;13(1):1-8.

(41) Derananian CA, Desai P, Smith-Ray R, Seymour RB, Hughes SL. Perceived versus actual factors associated with adoption and maintenance of an evidence-based physical activity program. *Transl Behav Med* 2012;2(2):209-17.

(42) Holden MA, Nicholls EE, Hay EM, Foster NE. Physical therapists' use of therapeutic exercise for patients with clinical knee osteoarthritis in the United Kingdom: in line with current recommendations? *Phys Ther* 2008;88(10):1109-21.

(43) Bedson J, Mottram S, Thomas E, Peat G. Knee pain and osteoarthritis in the general population: what influences patients to consult? *Fam Pract* 2007;24(5):443-53.

(44) Peabody JW, Luck J, Glassman P, Dresselhaus TR, Lee M. Comparison of vignettes, standardized patients, and chart abstraction: a prospective validation study of 3 methods for measuring quality. *JAMA* 2000;283(13):1715-22.

(45) Norcini J. Back to the future: clinical vignettes and the measurement of physician performance. *Ann Intern Med* 2004;141(10):813-4.

(46) Veloski J, Tai S, Evans AS, Nash DB. Clinical vignette-based surveys: a tool for assessing physician practice variation. *Am J Med Qual* 2005;20(3):151-7.

(47) Peabody JW, Liu A. A cross-national comparison of the quality of clinical care using vignettes. *Health Policy Plan* 2007;22(5):294-302.

(48) Conner M, Normal P. Predicting and changing health behaviour: research and practice with social cognition models. 3rd Ed. ed. Maidenhead: Open University Press; 2015.

(49) Khan KM, Weiler R, Blair SN. Prescribing exercise in primary care: ten practical steps on how to do it. *BMJ* 2011;343:806-7.

(50) Peterson JA. Get moving! Physical activity counseling in primary care. *J Am Acad Nurse Pract* 2007;19(7):349-57.

(51) Grandes G, Sanchez A, Sanchez-Pinilla RO, Torcal J, Montoya I, Lizarraga K, et al. Effectiveness of physical activity advice and prescription by physicians in routine primary care: a cluster randomized trial. *Arch Intern Med* 2009;169(7):694-701.

For peer review only - http://bmjopen.bmj.com/site/about/guidelines.xhtml

(52) Professional Associations for Physical Activity (Sweden). Physical activity in the prevention and treatment of disease. Sweden: Swedish National Institute of Public Health; 2010.

(53) Patel A, Schofield GM, Kolt GS, Keogh JW. General practitioners' views and experiences of counselling for physical activity through the New Zealand Green Prescription program. *BMC Fam Pract* 2011;12:119.

(54) Morden A, Jinks C, Ong BN, Porcheret M, Dziedzic KS. Acceptability of a 'guidebook' for the management of Osteoarthritis: a qualitative study of patient and clinician's perspectives. *BMC Musculoskelet Disord* 2014;15:427.

(55) Keele University, Arthritis Research UK, National Institute for Health Research. A guide for people who have osteoarthritis. 2014; Available at: http://www.keele.ac.uk/media/keeleuniversity/ri/primarycare/pdfs/OA_Guidebook.pdf. Accessed July/03, 2015.

(56) Arthritis Research UK. Osteoarthritis of the knee. Chesterfield. Available at: http://www.arthritisresearchuk.org/arthritis-information/conditions/osteoarthritis-of-the-knee.aspx: Arthritis Research UK; 2013.

(57) Gore M, Tai KS, Sadosky A, Leslie D, Stacey BR. Use and costs of prescription medications and alternative treatments in patients with osteoarthritis and chronic low back pain in community-based settings. *Pain Pract* 2012;12(7):550-60.

(58) Brand CA, Harrison C, Tropea J, Hinman RS, Britt H, Bennell K. Management of osteoarthritis in general practice in Australia. *Arthritis Care Res (Hoboken)* 2014;66(4):551-8.

(59) Wilkie R, Blagojevic-Bucknall M, Jordan KP, Lacey R, McBeth J. Reasons why multimorbidity increases the risk of participation restriction in older adults with lower extremity osteoarthritis: a prospective cohort study in primary care. *Arthritis Care Res (Hoboken*) 2013;65(6):910-9.

(60) Goodwin N, Curry N, Naylor C, Ross S, Duldig W. Managing people with long-term conditions. The Kings Fund 2010:98.

(61) Hay EM, Foster NE, Thomas E, Peat G, Phelan M, Yates HE, et al. Effectiveness of community physiotherapy and enhanced pharmacy review for knee pain in people aged over 55 presenting to primary care: pragmatic randomised trial. *BMJ* 2006 Nov 11;333(7576):995.

(62) Hurley MV, Walsh NE, Mitchell HL, Pimm TJ, Patel A, Williamson E, et al. Clinical effectiveness of a rehabilitation program integrating exercise, self-management, and active coping strategies for chronic knee pain: a cluster randomized trial. *Arthritis Rheum* 2007;57(7):1211-9.

(63) Dziedzic KS, Healey EL, Porcheret M, Ong BN, Main CJ, Jordan KP, et al. Implementing the NICE osteoarthritis guidelines: a mixed methods study and cluster randomised trial of a model osteoarthritis consultation in primary care--the Management of OsteoArthritis In Consultations (MOSAICS) study protocol. *Implement Sci* 2014;9(1):95.

(64) Bury TJ, Stokes EK. Direct access and patient/client self-referral to physiotherapy: a review of contemporary practice within the European Union. *Physiotherapy* 2013;99(4):285-91.

For peer review only - http://bmjopen.bmj.com/site/about/guidelines.xhtml

(65) NHS England. Technology Enabled Care Services (TECS). Launch of technology enabled care services (TECS) resource for commissioners. Available at: https://www.england.nhs.uk/ourwork/qual-clin-lead/tecs/. Accessed April/05, 2016.

(66) Rawstorn JC, Gant N, Direito A, Beckmann C, Maddison R. Telehealth exercise-based cardiac rehabilitation: a systematic review and meta-analysis. *Heart* 2016 Mar 2.

(67) Hoaas H, Andreassen HK, Lien LA, Hjalmarsen A, Zanaboni P. Adherence and factors affecting satisfaction in long-term telerehabilitation for patients with chronic obstructive pulmonary disease: a mixed methods study. *BMC Med Inform Decis Mak* 2016 Feb 25;16(1):26-016-0264-9.

FIGURES

Figure 1 Flow-chart summarising the exercise types and initiation methods used by GPs' for the vignette patient with CKP

Figure 2 Barriers to using exercise reported by GPs

For peer review only

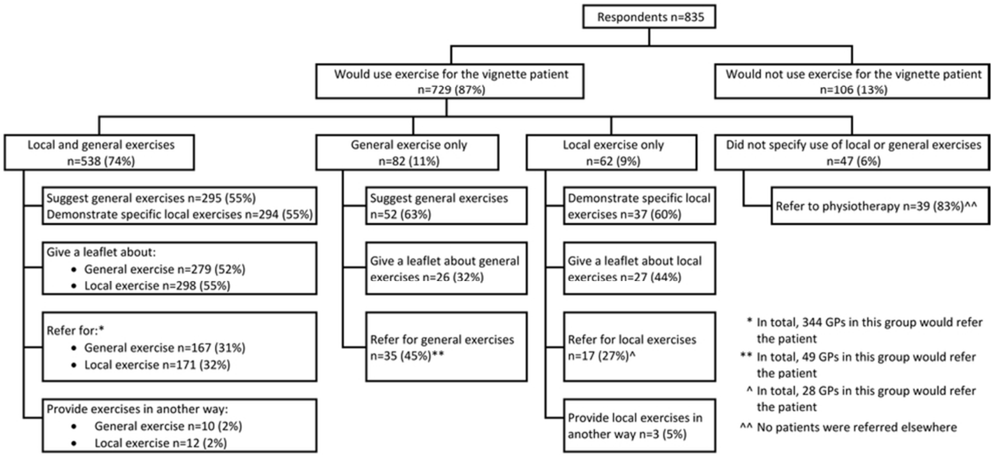

Figure 1 Flow-chart summarising the exercise types and initiation methods used by GPs' for the vignette patient with CKP

Figure 1

84x40mm (300 x 300 DPI)

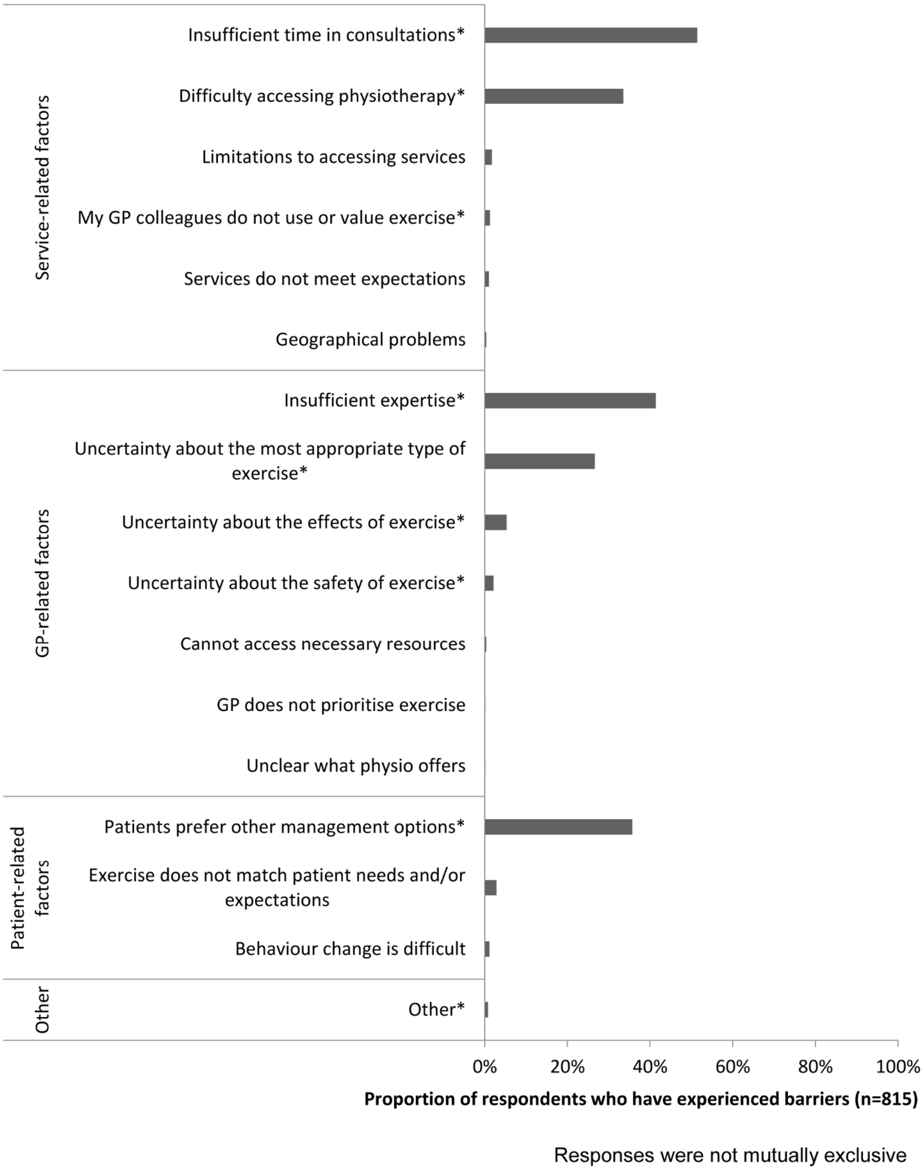

Figure 2 Barriers to using exercise reported by GPs
[asterisks denote barriers provided as a multiple response option in the questionnaire]
Figure 2
169x209mm (300 x 300 DPI)

Arthritis Research UK | primary care centre

Keele University

Management of Chronic Knee Pain Study

We are seeking the views of general practitioners who treat patients over 45 years old, with chronic knee pain.

If you **are not** a general practitioner, please tick this box and return the questionnaire without completing it any further ☐

If you **have not** managed someone with chronic knee pain in the last 6 months, please tick this box and return the questionnaire without completing it any further. ☐

This questionnaire should take no more than **20 minutes** to complete.

Return of your completed questionnaire will be interpreted as you providing your consent to participate in this study.

If you would like to participate in this study please either:

- Complete this paper version of the questionnaire and return it to Dr Elizabeth Cottrell, Academic Clinical Fellow GP Specialty Trainee, at the Arthritis Research UK Primary Care Centre, Keele University, Staffordshire, ST5 5BG using the enclosed FREEPOST envelope.
  OR
- Complete the online version of this questionnaire accessible at https://www.surveymonkey.com/s/Management_CKP

If you have any questions about this questionnaire or the study in general you can email Dr Elizabeth Cottrell at e.cottrell@keele.ac.uk

Instructions for completing this questionnaire

- When completing the questionnaire, please try and provide answers that most accurately reflect your usual clinical practice. There are no 'correct' or 'incorrect' answers.
- Where relevant please answer questions by ticking a box e.g. ☑
- Please do not consult any literature while completing this questionnaire.

Thank you for your help with this study

Unique survey ID	

For peer review only - http://bmjopen.bmj.com/site/about/guidelines.xhtml

Section 1: About you

1.1	Please state the year in which you qualified as a General Practitioner	☐
1.2	How many General Practitioners work in your practice (including yourself)?	☐
1.3	How do you best **describe yourself** (please tick **one box** only)<br>☐ GP Partner ☐ Salaried GP ☐ Locum GP<br>☐ Other, please specify ______	
1.4	Is your practice ☐ Urban ☐ Semi-rural ☐ Rural	
1.5	Are you ☐ Male ☐ Female	
1.6	Are you a **GP with a special interest (GPwSI)** in musculoskeletal conditions?<br>☐ Yes ☐ No	
1.7	Do you remember receiving any specific postgraduate musculoskeletal training which contained education about chronic knee pain? (By this we do not mean clinical placements or jobs in rheumatology or orthopaedics)<br>☐ Yes ☐ No	
1.8	Do you have, or have you ever suffered from chronic knee pain **yourself**?<br>☐ Yes ☐ No	

Section 2: Clinical scenario of a patient with chronic knee pain

Presented below is a clinical scenario of a patient **with chronic knee pain** who presents to you with this problem for the first time. All questions that follow relate to the care you would give this particular patient. Think about the patient's first consultation with you.

Patient: Mrs Jones, 58-year-old Prison Officer

History: First presentation of gradually worsening bilateral knee pain (right worse than left) over 2 years
No history of trauma
Pain always present when walking and at rest, worst when climbing stairs. No night pain.
Managing activities of daily living. Difficulty gardening.
Stopped going to gym – thinks was making pain worse
Only treatment tried is Ibuprofen once or twice when pain "really bad" – no benefit.
Came today finding work increasingly difficult due to the stairs
Usually well – no comorbidities

Medication: Nil

Examination: Body Mass Index 33
Knees – bilaterally no effusions. Joint tenderness upon palpation. Bilateral coarse crepitations.
Slightly reduced flexion of the right knee.
Hips – no abnormality detected

2.1 What **diagnosis** would you make at this point?

____

2.2 Using the words you would use with the patient, ***briefly*** state how you would **describe your diagnosis** to the patient

____

____

____

For peer review only - http://bmjopen.bmj.com/site/about/guidelines.xhtml

2.3 The patient's **symptoms** are: (please tick the **one** box that best reflects your opinion)

☐ Very severe ☐ Severe ☐ Moderate ☐ Mild ☐ Very mild

2.4 It is most likely that this patient's symptoms result from **knee damage** that is: (please tick the **one** box that best reflects your opinion)

☐ Very severe ☐ Severe ☐ Moderate ☐ Mild ☐ Very mild

2.5 Using the words you would use with the patient, ***briefly*** describe **what the future is likely to hold** with regards to her knee problem

____

____

2.6 What **investigation(s)/assessment(s)** would you do/order for this patient **at this point** and for what reason?

Please tick **all** you would undertake	Please indicate the reason for choosing this investigation (tick all that apply)					
	Reassure patient	To meet referral criteria	Confirm diagnosis	Inform treatment	Rule out other diagnoses	Inform prognosis
☐ None	---	---	---	---	---	---
☐ Blood tests	☐	☐	☐	☐	☐	☐
☐ Oxford knee score	☐	☐	☐	☐	☐	☐
☐ Knee x-ray	☐	☐	☐	☐	☐	☐
☐ Other - please state __________	☐	☐	☐	☐	☐	☐

2.7 **At this consultation**, what approaches would you use, or suggest, to manage this patient? (please tick **all** that apply)

☐ Ice ☐ Keep active ☐ Weak opioids (e.g. codeine) ☐ Topical NSAID

☐ Heat ☐ Provision of walking stick(s) ☐ COX II inhibitor ☐ Paracetamol

☐ Acupuncture ☐ Advice on footwear ☐ Antidepressants ☐ Glucosamine/ chondroitin

☐ Rest ☐ Exercise ☐ Injection of steroids ☐ Topical capsaicin

☐ Weight loss ☐ Bed rest ☐ Oral non-selective NSAIDs (e.g. Ibuprofen) ☐ Strong opioids (e.g. morphine, Tramadol)

☐ None ☐ Transcutaneous electrical nerve stimulation

☐ Other (please state)

____

For peer review only

If you ticked "Exercise" in question 2.7 please go on to answer question 2.8. If not, please move straight on to question 2.9

2.8 There are a variety of approaches that can be used to manage chronic knee pain in general practice.

Of the following different approaches, **which, if any, would you use for this patient at this point**?

a) General exercises or increasing physical activity

☐ I would use ⇨ If you ***would use*** this strategy, please give details about what you would **actually do** (please tick any that apply)

- ☐ Suggest general exercises ⇨ Please state type of exercise ____________
- ☐ Give a leaflet
- ☐ Refer
- ☐ Other ⇨ Please state what other actions you would do ____________

☐ I would **not** use ⇨ If you ***would not use*** the strategy ***but would like to***, please tell us **what prevents** you (please tick any that apply)

- ☐ Insufficient expertise
- ☐ Insufficient time
- ☐ Other ⇨ Please state what other reasons prevent you (e.g. patient factors, access difficulties, uncertainty of benefit etc) ____________

b) Local knee or quadriceps strengthening exercises

☐ I would use ⇨ If you ***would use*** this strategy**,** please give details about what you would **actually do** (please tick any that apply)

- ☐ Demonstrate specific exercises
- ☐ Give a leaflet
- ☐ Refer
- ☐ Other ⇨ Please state what other actions you would do ____________

☐ I would **not** use ⇨ If you ***would not use*** the strategy ***but would like to***, please tell us **what prevents** you (please tick any that apply)

- ☐ Insufficient expertise
- ☐ Insufficient time
- ☐ Other ⇨ Please state what other reasons prevent you (e.g. patient factors, access difficulties, uncertainty of benefit etc) ____________

c) Follow up patient to check to see if she is undertaking exercise on a regular basis

☐ I would use ⇨ If you ***would use*** this strategy**,** please give details about what you would **actually do** (please tick any that apply)

- ☐ Planned follow-up ⇨
  - When: Please define number of days/weeks/months ____________
  - How: ☐ Face to face ☐ Via telephone
  - With whom: ☐ Yourself ☐ Another professional or service – please state ____________
- ☐ Opportunistic follow-up ⇨
  - When: ☐ If patient fails to improve ☐ Check when patient is next seen with this or any problem
- ☐ Other ⇨ Please state what other actions you would do ____________

☐ I would **not** use ⇨ If you ***would not use*** the strategy ***but would like to***, please tell us **what prevents** you (please tick any that apply)

- ☐ Insufficient expertise
- ☐ Insufficient time
- ☐ Other ⇨ Please state what other reasons prevent you ____________

For peer review only - http://bmjopen.bmj.com/site/about/guidelines.xhtml

2.9 Would you refer the patient to see someone else, either in the primary or community team or into secondary care, at this point? ☐ Yes ☐ No

If yes, to whom would you refer her?

____

2.10 Do you usually provide **written information** for patients in this situation? ☐ Yes ☐ No

If yes, please state the source of your written information

☐ Patient.co.uk or Emis Mentor ☐ Arthritis Research UK (previously known as ARC) or Arthritis Care ☐ Other, please state __________________

If you would provide written information, it would be very helpful to see a copy by returning it in the freepost envelope provided (**please write on your unique survey ID**) or by providing us with the link for any online information you access.

We are interested in your clinical opinion about patients **aged 45 years and over** with **chronic knee pain**. In this age group chronic knee pain is almost always due to knee osteoarthritis. Please answer all of the following questions using the definition of chronic knee pain as follows: knee pain and associated symptoms that have been present for more than 3 months not resulting from a fracture, infection, systemic rheumatological problem, metastases or surgery.

Section 3: Chronic knee pain in general practice

		I have never heard about or read it	I have heard of it but not seen it	I have seen it but not read it	I have read the full guideline and/or summary	I have read and consider the guideline when planning management
3.1	How much have you heard about or read the guideline published by NICE in 2008 for the care and management of osteoarthritis in adults? (please tick **one** box)	☐	☐	☐	☐	☐

Please consider **your role** in managing patients aged over 45 years old with chronic knee pain. Please indicate the extent to which you agree or disagree with the statements given by ticking **one** box per row.

		Strongly disagree	Disagree	Neither agree or disagree	Agree	Strongly agree
3.2	It is part of my job to manage people with chronic knee pain	☐	☐	☐	☐	☐
3.3	I have enough time to manage patients with chronic knee pain	☐	☐	☐	☐	☐
3.4	Managing patients with chronic knee pain is a priority for me	☐	☐	☐	☐	☐
3.5	Managing patients with chronic knee pain is of clinical interest to me	☐	☐	☐	☐	☐
3.6	It is part of my job to reassure patients about the safety of exercise for chronic knee pain	☐	☐	☐	☐	☐
3.7	It is part of my job to provide patients with chronic knee pain with a written management plan	☐	☐	☐	☐	☐

For peer review only

		I have no role in including exercise in the management	I inform patients that exercise is a management option	I advise patients to use exercise to manage their knee pain	I recommend the types of exercise patients could use	I give information on the type, frequency and duration of specific exercises
3.8	Which statement best describes your role in including exercise in the management plan of a patient with chronic knee pain? (please tick **one** box)	☐	☐	☐	☐	☐

3.9 We are interested to hear about **your experiences of barriers which might prevent the use of exercise** in your management of chronic knee pain. Please tick **all** of the barriers that you have experienced

- ☐ Insufficient time in consultations
- ☐ Insufficient expertise to give detailed information
- ☐ Difficulty accessing physiotherapy
- ☐ Uncertainty about the effects of exercise
- ☐ Patients prefer other management options
- ☐ My GP colleagues do not use or value exercise
- ☐ Uncertainty about the most appropriate type of exercise
- ☐ Uncertainty about the safety of exercise
- ☐ Other (please state) ____________________

____

Section 4: Your views about chronic knee pain

Below is a list of **possible causes** for a patient developing chronic knee pain. Please indicate the extent to which you agree or disagree with these causes by ticking **one** box in each row.

		Strongly disagree	Disagree	Neither agree or disagree	Agree	Strongly agree
4.1	Hereditary/runs in the family	☐	☐	☐	☐	☐
4.2	Being overweight/obese	☐	☐	☐	☐	☐
4.3	A person's own mental attitude e.g. thinking about life negatively	☐	☐	☐	☐	☐
4.4	A person's emotional state e.g. feeling down, anxious	☐	☐	☐	☐	☐
4.5	Ageing	☐	☐	☐	☐	☐
4.6	Accident or injury	☐	☐	☐	☐	☐
4.7	Manual work	☐	☐	☐	☐	☐
4.8	Sport	☐	☐	☐	☐	☐
4.9	Osteoarthritis	☐	☐	☐	☐	☐
4.10	Changes consistent with osteoarthritis seen on x-ray	☐	☐	☐	☐	☐

For peer review only

When completing the following questions, please consider patients **aged over 45 years old** with **chronic knee pain**. Please indicate the extent to which you agree or disagree with the statements given by ticking one box per row.

		Totally disagree	Largely disagree	Disagree to some extent	Agree to some extent	Largely agree	Totally agree
4.11	Mental stress can cause chronic knee pain even in the absence of tissue damage	☐	☐	☐	☐	☐	☐
4.12	The cause of chronic knee pain is unknown	☐	☐	☐	☐	☐	☐
4.13	Pain is a nociceptive stimulus, indicating tissue damage	☐	☐	☐	☐	☐	☐
4.14	A patient suffering from severe chronic knee pain will benefit from physical exercise	☐	☐	☐	☐	☐	☐
4.15	Functional limitations associated with chronic knee pain are the result of psychosocial factors	☐	☐	☐	☐	☐	☐
4.16	Patients with chronic knee pain should preferably practice only pain free movements	☐	☐	☐	☐	☐	☐
4.17	Therapy may have been successful even if pain remains	☐	☐	☐	☐	☐	☐
4.18	Chronic knee pain indicates the presence of organic injury	☐	☐	☐	☐	☐	☐
4.19	If chronic knee pain increases in severity, I immediately adjust the intensity of my treatment accordingly	☐	☐	☐	☐	☐	☐
4.20	If therapy does not result in a reduction in chronic knee pain, there is a high risk of severe restrictions in the long term	☐	☐	☐	☐	☐	☐
4.21	Pain reduction is a precondition for the restoration of normal functioning	☐	☐	☐	☐	☐	☐
4.22	Increased pain indicates new tissue damage or the spread of existing damage	☐	☐	☐	☐	☐	☐
4.23	There is no effective treatment to eliminate chronic knee pain	☐	☐	☐	☐	☐	☐
4.24	Even if the pain has worsened, the intensity of the next treatment can be increased	☐	☐	☐	☐	☐	☐
4.25	If patients complain of pain during exercise, I worry that damage is being caused	☐	☐	☐	☐	☐	☐
4.26	The severity of tissue damage determines the level of pain	☐	☐	☐	☐	☐	☐
4.27	Learning to cope with stress promotes recovery from chronic knee pain	☐	☐	☐	☐	☐	☐
4.28	Exercises that may be knee straining should not be avoided	☐	☐	☐	☐	☐	☐
4.29	In the long run, patients with chronic knee pain have a higher risk of developing severe functional impairments	☐	☐	☐	☐	☐	☐

For peer review only - http://bmjopen.bmj.com/site/about/guidelines.xhtml

Section 5: Your views about the role of exercise in treating chronic knee pain

We are interested in your views about the **role of exercise** in the treatment of **chronic knee pain in patients over 45 years old**. Please indicate the extent to which you agree or disagree with the statements given by ticking one box per row.

		Strongly disagree	Disagree	Neither agree or disagree	Agree	Strongly agree
5.1	GPs should prescribe quadriceps strengthening exercises to every patient with chronic knee pain	☐	☐	☐	☐	☐
5.2	GPs should prescribe general exercise, for example, walking or swimming, for every patient with chronic knee pain	☐	☐	☐	☐	☐
5.3	Knee problems are improved by quadriceps strengthening exercises	☐	☐	☐	☐	☐
5.4	Knee problems are improved by general exercise, for example walking or swimming	☐	☐	☐	☐	☐
5.5	Quadriceps strengthening exercises for the knee are safe for everybody to do	☐	☐	☐	☐	☐
5.6	General exercise, for example walking or swimming is safe for everybody to do	☐	☐	☐	☐	☐
5.7	Exercise for chronic knee pain is most beneficial when it is tailored to meet individual patient needs	☐	☐	☐	☐	☐
5.8	A standard set of exercises is sufficient for every patient with chronic knee problems	☐	☐	☐	☐	☐
5.9	GPs should educate chronic knee pain patients about how to change their lifestyle for the better	☐	☐	☐	☐	☐
5.10	It is important that people with chronic knee pain increase their overall activity levels	☐	☐	☐	☐	☐
5.11	How well a patient complies with their exercise programme determines how effective it will be	☐	☐	☐	☐	☐
5.12	GPs should follow up patients to monitor extent of continuation of exercises	☐	☐	☐	☐	☐
5.13	It is the patient's own responsibility to continue doing their exercise programme	☐	☐	☐	☐	☐
5.14	Exercise is effective for patients if an x-ray shows severe knee osteoarthritis	☐	☐	☐	☐	☐
5.15	Exercise works just as well for everybody, regardless of the amount of pain they have	☐	☐	☐	☐	☐
5.16	Increasing the strength of the muscles around the knee stops the knee problem getting worse	☐	☐	☐	☐	☐
5.17	Increasing overall activity levels stops the knee problem getting worse	☐	☐	☐	☐	☐
5.18	Exercise for chronic knee pain is more effectively provided by physiotherapists than GPs	☐	☐	☐	☐	☐
5.19	Time constraints prevent GPs from providing advice on individual exercises for chronic knee pain	☐	☐	☐	☐	☐
5.20	Exercise for chronic knee pain should preferably be used after drug treatment has been tried	☐	☐	☐	☐	☐
5.21	Exercise for chronic knee pain would be used more frequently if access to physiotherapy was easier	☐	☐	☐	☐	☐

For peer review only - http://bmjopen.bmj.com/site/about/guidelines.xhtml

Would you be happy for us to contact you again in the future regarding this study?

☐ Yes ☐ No

If you answered YES to the question above please provide your name and contact details below (these details will be kept separately from your responses to the questionnaire):

**Name:**	
**Daytime telephone number:**	
**Address:**	
**Email:**	

End of Questionnaire

You have reached the end of the questionnaire. Please return the questionnaire in the **FREEPOST** envelope provided.

If you have any questions about this questionnaire or the study in general, you can email Dr Elizabeth Cottrell at e.cottrell@keele.ac.uk

Thank you for taking the time to complete this questionnaire. Your time and participation is greatly appreciated.

Unique survey ID

For peer review only

BMJ Open

GP attitudes, beliefs and behaviours regarding exercise for chronic knee pain: a questionnaire survey

Journal:	*BMJ Open*
Manuscript ID	bmjopen-2016-014999.R1
Article Type:	Research
Date Submitted by the Author:	22-Mar-2017
Complete List of Authors:	Cottrell, Elizabeth ; Keele University, Research Institute for Primary Care & Health Sciences<br>Foster, Nadine; Keele University, Arthritis Research UK Primary Care Centre, Research Institute of Primary Care and Health Sciences; Keele University, Keele Clinical Trials Unit<br>Porcheret, Mark; Keele University, Arthritis Research UK Primary Care Centre, Research Institute of Primary Care and Health Sciences<br>Rathod, Trishna; Keele University, Arthritis Research UK Primary Care Centre, Research Institute of Primary Care and Health Sciences; Keele University, Keele Clinical Trials Unit<br>Roddy, Edward; Keele University, Arthritis Research UK Primary Care Centre, Research Institute of Primary Care and Health Sciences
**Primary Subject Heading**:	General practice / Family practice
Secondary Subject Heading:	Evidence based practice, Rheumatology
Keywords:	Knee < ORTHOPAEDIC & TRAUMA SURGERY, osteoarthritis, exercise, general practitioners, surveys and questionnaires, attitude

SCHOLARONE™
Manuscripts

GP attitudes, beliefs and behaviours regarding exercise for chronic knee pain: a questionnaire survey

Elizabeth Cottrell MBChB(Hons), MRCGP, Ph.D.*^, Nadine E. Foster BSc(Hons), DPhil, FCSP*†, Mark Porcheret MBBS, FRCGP, Ph.D.*, Trishna Rathod MSc*†, Edward Roddy DM, FRCP*

*Arthritis Research UK Primary Care Centre, Research Institute for Primary Care and Health Sciences, Keele University, UK

†Keele Clinical Trials Unit, David Weatherall Building, Keele University, Staffordshire, ST5 5BG^Corresponding author: Arthritis Research UK Primary Care Centre, Research Institute for Primary Care and Health Sciences, Keele University, Keele, Staffordshire, ST5 5BG.

Telephone: 01782 734870. Fax: 01782 734719. Email: e.cottrell@keele.ac.uk

KEY WORDS

Chronic knee pain, osteoarthritis, exercise, general practitioners, surveys and questionnaires, attitude

Word count excluding title page, abstract, references, tables and figures: 2862

Number of tables and figures: Figures 2, Tables 4

For peer review only

ABSTRACT

Objectives:

The aim of this study was to investigate general practitioners' (GPs) attitudes, beliefs and behaviours regarding the use of exercise for patients with chronic knee pain (CKP) attributable to osteoarthritis.

Setting:

Primary care GPs in the UK.

Participants:

5000 GPs, randomly selected from Binley's database, were mailed a cross-sectional questionnaire survey.

Outcome measures:

GPs' attitudes and beliefs were investigated using attitude statements, and reported behaviours were identified using vignette-based questions. GPs were invited to report barriers experienced when initiating exercise with patients with CKP.

Results:

835 (17%) GPs responded. Overall, GPs were positive about general exercise for CKP. 729 (87%) reported using exercise, of which, 538 (74%) reported that they would use both general and local (lower limb) exercises. However, only 92 (11% of all responding) GPs reported initiating exercise in ways aligning with best-evidence recommendations. 815 (98%) GPs reported barriers in using exercise for patients with CKP, most commonly, insufficient time in consultations (n=419; 51%) and insufficient expertise (n=337; 41%).

Conclusions:

For peer review only

While GPs' attitudes and beliefs regarding exercise for CKP were generally positive, initiation of exercise was often poorly aligned with current recommendations, and barriers and uncertainties were reported. GPs' use of exercise may be improved by addressing the key barriers of time and expertise, by developing a pragmatic approach that supports GPs to initiate individualised exercise, and/or by other professionals taking on this role.

STRENGTHS AND LIMITATIONS OF THIS STUDY

- This large UK survey is the first known to directly, specifically and concurrently investigate the attitudes, beliefs and behaviours of GPs regarding exercise for patients with CKP. Exercise initiation did not concur with best-evidence recommendations and GPs reported uncertainties and barriers in relation to using exercise.
- The questionnaire was pre-tested and piloted before being used in this main study. Use of a vignette to investigate clinical management ensured a consistent patient scenario to all GPs and minimised the confounders inherent in observational research using real patients.
- Limitations of this study include the likely over-estimation of exercise use among GPs given the low response rate (response bias), the self-report nature of the questionnaire (social desirability bias), inability to explore underlying reasons for responses and the relatively uncomplicated vignette case. However, as GPs should be using exercise for all patients with CKP, the results of this survey are valuable for indicating an apparent evidence-practice gap in the way in which GPs employ exercise with this patient group.

For peer review only - http://bmjopen.bmj.com/site/about/guidelines.xhtml

ABBREVIATIONS

CI = confidence interval; CKP = chronic knee pain; EULAR = European League Against Rheumatism; GP = general practitioners; HCP = healthcare professional; MDS = minimum data set; NICE = National Institute for Health and Care Excellence; OA = osteoarthritis; OARSI = Osteoarthritis Research Society International; OR = odds ratio; UK = United Kingdom

For peer review only

INTRODUCTION

General practitioners (GPs) are the most frequently accessed source of formal medical advice and treatment for patients in the UK with chronic knee pain (CKP).[1-3] CKP is defined in this study as being synonymous with clinical knee osteoarthritis (OA),[4] that is; mechanical knee pain, with or without loss of function, and with or without radiographic changes consistent with OA, that has lasted for at least three months in people aged 45 years and older,[5] and for which an alternative diagnosis is unlikely.[4] Globally, OA is among the leading causes of years lived with disability,[6] thus it is unsurprising that CKP is a common presentation to GPs.[7,8] Exercise, comprising of both local (lower limb focused) and general (aerobic) exercise, is recommended as core treatment for CKP,[4,9] its provision is one of the eight UK OA quality standards[10] and international OA experts recognise provision of information about regular physical activity and individualised exercise to patients as essential.[11] Empirical research evidence now unequivocally demonstrates that general aerobic, local strengthening and flexibility exercises improve pain and function in patients with CKP.[12] In line with wider self-management strategies, best practice outlined by the National Institute for Health and Care Excellence (NICE) OA guidelines with regards to integrating exercise into the management of patients with CKP involves providing verbal advice about both general and local exercise (which should be specific and individualised[4,13]) supported with written information.[4] Where GPs feel unable to provide specific and individualised advice, referral of patients to appropriate exercise specialists (e.g. physiotherapist) would be appropriate. While it is recognised that delivery of care for CKP is multidisciplinary, the exact roles and explicit expectations of GPs (and other professionals) regarding the delivery of core management approaches is not provided within current guidelines. This could have the consequence that no professional undertakes certain activities in the belief that others will.

To maximise patient outcomes, GPs should align their management with best-evidence recommendations. As sociocognitive behavioural theories suggest an association between

For peer review only - http://bmjopen.bmj.com/site/about/guidelines.xhtml

individuals' attitudes and beliefs and their behaviours,[14-16] concurrent investigation of attitudes, beliefs and behaviours of GPs was undertaken. A systematic review revealed a paucity of data specifically examining GPs use of exercise for patients with CKP, however attitudes regarding exercise were variable, it appeared to be underused and its implementation by GPs was unclear.[5] The role that GPs perceive themselves to have in delivering these management approaches was also not clear. The aim of this cross-sectional questionnaire survey was to identify the attitudes, beliefs and behaviours of UK GPs regarding the use of exercise for patients with CKP. Analysis of factors associated with the use of exercise among this group have been published elsewhere.[17]

METHODS

A cross-sectional survey was used to investigate GPs attitudes, beliefs and behaviours regarding exercise for CKP. The survey tool had previously been developed through pre-testing by a local group of GPs and a subsequent pilot study with 172 UK GPs,[18] which was designed to investigate the likely response to the questionnaire, to finalise the survey tool and to test methods to maximise quantity and quality of responses.

In this main study, GPs were mailed an eight-page questionnaire (see Supplementary File 1), a cover letter and a postage-paid reply envelope in January 2014. Non-responders were sent a reminder postcard after two weeks and, four weeks after the initial mailing, persistent non-responders were mailed a second copy of the questionnaire with a cover letter and postage-paid reply envelope. At each stage non-responders who did not wish to complete the full questionnaire were invited to provide minimum data sets (MDS; gender, year of qualification, practice size and setting) and a reason for non-response. Attitude statements associated with a five-point Likert scale explored GPs' attitudes and beliefs about exercise for CKP. These were minimally adapted from the work of Holden and colleagues who investigated this among physiotherapists[19] and older adults with CKP[20] and were derived from the MOVE consensus recommendations, designed to help healthcare

For peer review only

professionals (HCPs) to initiate exercise in the management of a patient with lower limb OA[21]. GPs' reported clinical behaviour was investigated using multiple response questions associated with a vignette case (see Table 1). GPs reporting to use exercise were requested to indicate the type of exercise and how this was initiated. A multiple response item, with space for free-text, investigated GPs' experiences of barriers to using exercise for CKP. Completion and return of the questionnaire by the GP was interpreted as consent to participate in the study.

Table 1 Vignette used in the questionnaire to assess GPs' reported behaviours

**Patient:**	Mrs Jones, 58-year-old Prison Officer
**History:**	First presentation of gradually worsening bilateral knee pain (right worse than left) over 2 years<br>No history of trauma<br>Pain always present when walking and at rest, worst when climbing stairs. No night pain.<br>Managing activities of daily living. Difficulty gardening.<br>Stopped going to gym – thinks was making pain worse<br>Only treatment tried is ibuprofen once or twice when pain "really bad" – no benefit.<br>Came today finding work increasingly difficult due to the stairs<br>Usually well – no comorbidities
**Medication:**	Nil
**Examination:**	Body Mass Index 33<br>Knees – bilaterally no effusions. Joint tenderness upon palpation. Bilateral coarse crepitations.<br>Slightly reduced flexion of the right knee.<br>Hips **–** no abnormality detected

A minimum sample size of 288 responding GPs was required to estimate the use of exercise, based on a conservative estimate of 75% reporting exercise use informed by the pilot study[18] and a margin of error of <5%.[22] After increasing the minimum sample size to adjust for other planned regression analyses (exploring associations between reported exercise use and attitudes/beliefs (paper in preparation)) and anticipating a response rate of 20%,[18] 5000 UK GPs were randomly selected from Binley's database; a database containing the contact details of GPs in the UK which is updated quarterly. Binley's extracted a simple random sample of GPs from their database and removed and replaced any GPs included in the sample used for the previous pilot study. Study exclusion criteria were not being a GP and not having managed someone with CKP in the previous six months.

For peer review only

GPs' practice postcodes from each UK country were transformed into their corresponding Index of Multiple Deprivation rank[23-26] and split into quintiles (1=most deprived, 5=least deprived). Responses to the GP attitude statements were condensed into three categories ((strongly) disagree, neither disagree nor agree, and (strongly) agree) and free-text responses (associated "other" responses and regarding type of exercise the GPs would use) underwent thematic analysis, categorising responses into pre-defined categories that emerged from responses to the pilot study and developing new categories as appropriate, before commencing descriptive analyses. Responses to GP attitude statements were interpreted as follows: unamity = 100%, consensus = 75-99%, majority view = 51-74%, no consensus = 0-50%[19,27]. To assess for possible response bias in questionnaire respondents versus MDS responders, demographic data of each type of responders were compared using logistic regression to obtain unadjusted odds ratio with 95% confidence interval (CI; gender, practice area deprivation and practice type) and mean difference with 95% CI (mean years since qualification and mean number of GPs in respondents practice). An *a posteriori* analysis was undertaken due to the timing of the main survey coinciding with publication of the revised version of the NICE OA guidelines on 12th February 2014 (four weeks after the baseline mailing of the survey). To establish whether the publication of these guidelines, and the publicity associated with this event had an impact on the proportion of GPs using exercise, logistic regression was used to compare the use of exercise among responses received before the date of publication of the revised guideline with those responses received after. All analyses were performed using IBM SPSS Statistics (Version 20).

RESULTS

Response

Of the 5000 GPs sent the questionnaire, 58 responders met one or more exclusion criteria and 835 returned a completed questionnaire (adjusted response 17%). A further 470

For peer review only

provided MDS. The most common reason for returning MDS, rather than a full response was, having insufficient time (n=408, 87%). The characteristics of GPs responding with a MDS were similar to those responding with a full questionnaire, except they had been qualified for longer and were more likely to work in practices in the most deprived areas (Table 2). When compared to GPs with practice postcodes in the mid-deprived quintile (OR (95% CI)), those in the most deprived (0.72 (0.60,0.87)) and second most deprived (0.76 (0.62,0.92)) were significantly less likely to respond in any way (completed questionnaire or MDS), although the absolute difference in the proportions responding were small.

Table 2 Demographic details of questionnaire respondents versus those providing minimum data sets

Variable	Category	Response type		OR/mean difference (95% CI)
		MDS (n=470)	Completed questionnaire (n=835)	
**Gender**	Male	247 (53%)	401 (49%)	OR 1.00
	Female	219 (47%)	417 (51%)	OR 1.17 (0.93,1.47)
**Practice area deprivation**	Most deprived	121 (26%)	181 (22%)	**OR 0.63 (0.45,0.89)**
	Second most deprived	106 (23%)	156 (19%)	**OR 0.62 (0.44,0.88)**
	Mid-deprived	85 (18%)	202 (24%)	OR 1.00
	Second least deprived	84 (18%)	160 (19%)	OR 0.80 (0.56,1.16)
	Least deprived	73 (16%)	135 (16%)	OR 0.78 (0.53,1.14)
**Practice type**	Urban	254 (56%)	449 (54%)	OR 1.00
	Semi-rural	155 (34%)	275 (33%)	OR 1.00 (0.78,1.29)
	Rural	43 (10%)	103 (13%)	OR 1.36 (0.92,2.00)
**Mean (SD) years since qualification**		21.64 (10.03)	18.40 (10.33)	Mean difference = **-3.24 (-2.06,-4.42)**
**Mean (SD) number of GPs in respondent's practice**		6.44 (3.67)	6.44 (3.20)	Mean difference = <0.01 (-0.38,0.39)
**Information only requested in questionnaire**				
**Type of GP**	GP partner	---	656 (79%)	---
	Salaried GP	---	151 (18%)	
	Locum GP	---	20 (2%)	
	Other	---	5 (1%)	
**GP with special interest in musculoskeletal conditions**		---	50 (6%)	---
**Received postgraduate education about CKP**		---	319 (39%)	---
**Personal experience of CKP**		----	166 (20%)	---
Maximum missing data for any cell was 6%. CI = confidence interval; CKP = chronic knee pain, GP = general practitioner; MDS = minimum data set; OR = odds ratio; SD = standard deviation				

For peer review only

Attitudes of GPs regarding exercise for CKP

Table 3 summarises responses to GP exercise attitude statements, none were unanimous. Generally, GPs were more positive about general exercise than local exercise, particularly with regards to safety and efficacy. However, more GPs agreed that increasing the strength of the muscles around the knee stops the knee problem getting worse compared with those who agreed that increasing overall physical activity would do the same. No consensus was reached regarding the statement '*exercise works just as well for everybody, regardless of the amount of pain they have*', however the greatest proportion of respondents disagreed. GPs recognised the need to tailor exercises to individual patients, acknowledged the importance of adherence with exercise but placed responsibility for adherence on the patient.

For peer review only

Table 3 Responses to GP attitude statements derived from the MOVE consensus recommendations [21]

MOVE consensus proposition	Attitude statement	(Strongly) disagree	Neither disagree or agree	(Strongly) agree
**Items relating to the benefits of exercise (number of respondents)**				
Prescription of both general (aerobic fitness training) and local (strengthening) exercises is an essential, core aspect of management for every patient with hip or knee OA	GPs should prescribe quadriceps strengthening exercises to every patient with CKP (n=822)	8%	22%	69%
	GPs should prescribe general exercise, for example, walking or swimming, for every patient with CKP (n=824)	3%	8%	89%
Both strengthening and aerobic exercise can reduce pain and improve function and health status in patients with knee and hip OA	Knee problems are improved by quadriceps strengthening exercises (n=824)	<1%	11%	88%
	Knee problems are improved by general exercise, for example, walking or swimming (n=824)	1%	7%	93%
There are few contraindications to the prescription of strengthening or aerobic exercise in patients with hip or knee OA	Quadriceps strengthening exercises for the knee are safe for everybody to do (n=821)	15%	30%	56%
	General exercise, for example, walking or swimming, is safe for everybody to do (n=820)	13%	16%	71%
	Exercise works just as well for everybody, regardless of the amount of pain they have (n=823)	49%	29%	22%
The effectiveness of exercise is independent of the presence or severity of radiographic findings	Exercise is effective for patients if an x-ray shows severe knee osteoarthritis (n=822)	16%	32%	52%
Improvements in muscle strength and proprioception gained from exercise programmes may reduce the progression of knee and hip OA	Increasing the strength of the muscles around the knee stops the knee problem getting worse (n=824)	16%	29%	55%
	Increasing the overall activity levels stops the knee problem getting worse (n=822)	19%	38%	43%
**Items relating to the delivery of, and adherence to, exercise (number of respondents)**				
Exercise therapy for OA of the hip or knee should be individualised and patient-centred taking into account factors such as age, comorbidity and overall mobility	Exercise for CKP is most beneficial when it is tailored to meet individual patient needs (n=823)	1%	9%	90%
	A standard set of exercises is sufficient for every patient with chronic knee problems (n=821)	51%	36%	13%
**To be** effective, exercise programmes should include…advice and education to promote a positive lifestyle change with an increase in physical activity	GPs should educate CKP patients about how to change their lifestyle for the better (n=823)	1%	6%	93%
	It is important that people with CKP increase their overall activity levels (n=824)	1%	10%	89%

For peer review only

Adherence is the principal predictor of long-term outcome from exercise in patients with knee or hip OA	How well a patient complies with their exercise programme determines how effective it will be (n=825)	3%	11%	86%
Strategies to improve and maintain adherence should be adopted, e.g. long-term monitoring/review and inclusion of spouse/family in exercise	GPs should follow-up patients to monitor extent of continuation of exercises (n=823)	30%	37%	34%
	It is the patient's own responsibility to continue doing their exercise programme (n=826)	1%	6%	93%

Consensus categorised according to: unamity = 100%, consensus = 75-99%, majority view = 51-74%, no consensus = 0-50% (19,27). CKP = chronic knee pain; GP = general practitioner. Maximum missing data for any item was 2%.

For peer review only

GPs' reported use of exercise for CKP

Of the 835 respondents, 729 (87%) reported using exercise of some type for the vignette case. Figure 1 summarises the types of exercise and initiation methods that GPs reported that they would use. Among GPs reporting to suggest general exercise (n=347), the most common recommendations were swimming (49%), walking (41%) and cycling (34%). Only 17 (5%) GPs explicitly stated that general exercise should be tailored to patient's abilities and/or interests. Among GPs reporting to use exercise, 413 (57%) stated they would achieve this by referring the patient to a physiotherapist. Table 4 cross-tabulates the exercise initiation strategies GPs reported to use for both general and local exercise and shows the most common combinations of approaches were *suggesting* general exercise and *demonstrating* local exercise, and giving the patient a leaflet about both exercise types. Thirty-two (6%) GPs reporting to use both exercise types stated they would achieve this solely by referring the patient to another HCP. Ninety-two GPs (17% of those using both local and general exercise, 11% of all respondents) reported to use strategies aligned with evidence-based recommendations;[4] they advised, or referred for, local and general exercise and provided written information for both exercise types (Table 4). The use of exercise was not significantly different among responses received after the publication of the revised NICE OA guidelines (273/314, 87%) when compared with those received before (456/521, 88%; OR 0.95 (95% CI 0.62,1.44)).

[INSERT Figure 1 Flow-chart summarising the exercise types and initiation methods used by GPs' for the vignette patient with CKP]

For peer review only - http://bmjopen.bmj.com/site/about/guidelines.xhtml

Table 4 Methods used to initiate local and general exercise by GPs using both exercise types

**Methods used to include general exercises**	**Methods used to include local exercises**			
	Does not demonstrate, give leaflet nor refer	***Refers and/or demonstrates***	***Leaflet only***	***Leaflet and demonstrates, and/or refer***
Does not suggest, give leaflet nor refer	0%	1%	0%	<1%
Refers and/or suggests only	<1%	33%	6%	7%
Leaflet only	0%	4%	11%	7%
Leaflet, suggests and/or refers	<1%	6%	6%	17%

Management strategies used by >5% GPs are emboldened. The responses in the box are those that are consistent with evidence-based recommendations (i.e. providing advice and written information about both types of exercise and/or referring if needed). n=535

For peer review only

Use of follow-up

Of the 729 GPs reporting to use of exercise, 494 (68%) stated that they would follow-up the vignette patient to establish ongoing engagement with regular exercise. This was most commonly achieved through opportunistic follow-up (n=303, 61%) which most GPs (n=253, 84%) suggested would occur if the vignette patient failed to improve and re-consulted.

Barriers to exercise use

Most GPs (n=815, 98%) reported having experienced barriers when using exercises for patients with CKP which included: 1) service-related, 2) GP-related and 3) (perceived) patient-related barriers (Figure 2). The most frequently reported barriers were insufficient time (n=419, 51%), insufficient expertise (n=337, 41%) and the perception that patients prefer other management options (n=291, 36%).

[INSERT Figure 2 Barriers to using exercise reported by GPs]

DISCUSSION

Summary

This cross-sectional questionnaire survey sought to identify the attitudes, beliefs and behaviours of UK GPs regarding the use of exercise for patients with CKP. While most GPs

For peer review only - http://bmjopen.bmj.com/site/about/guidelines.xhtml

agreed that they should *'prescribe'* local and general exercise to all patients with CKP, believed that CKP is improved by local and general exercise, and reported that they would use exercise in the management of the vignette patient, only a tenth of responding GPs reported initiating exercise in a way that matches best-evidence recommendations.[4] This evidence-practice gap is perhaps unsurprising given that most GPs reported key barriers of time and expertise. A small number of GPs reported relying solely on referring the vignette patient to another HCP for both exercise types. This approach delays commencement of exercise as patients are not equipped to start exercising immediately after the consultation with the GP, relies on the receiving HCP delivering best practice and may not represent efficient use of services, if this is representative of GPs' usual approach for patients with CKP.

Comparison with existing literature

Variable GP attitudes regarding exercise for CKP were recognised in an earlier systematic review[5] and the uncertainty regarding exercise efficacy identified by the current study supports this. GPs' responses to the MOVE attitude statements from the current study were compared to those from physiotherapists[19] and older adults with CKP.[20] In contrast to GPs being generally more positive regarding general exercise when compared to local exercise, physiotherapists were generally more positive about the safety and efficacy of local exercise,[19] and older adults with CKP reported low levels of agreement about the safety and efficacy of both types of exercise.[20] Although GPs were more positive about the efficacy and safety of both exercise types than either physiotherapists or those with CKP, the timing of the respective surveys must be considered. In the time between the previous and current surveys, new and/or revised versions of NICE,[4] Osteoarthritis Research Society International (OARSI)[9] and European League Against Rheumatism (EULAR)[28] guideline recommendations for OA have been published; thus familiarity with best-evidence recommendations may have increased.

For peer review only - http://bmjopen.bmj.com/site/about/guidelines.xhtml

GPs' reported behaviour was consistent with higher estimates from other physician questionnaire studies; between 9-89% of GPs advising exercise[5,29-31] and 10-77% referring patients with CKP to physiotherapy[5,29-31]. Given that estimates of GPs' exercise use are similar internationally, for example, advice to exercise was 46-76% in the UK[32,33] and 12-59% in the USA[31,34-37], the results are likely to be relevant beyond the UK. While the most common general exercise suggestions (swimming, walking and cycling) were clinically appropriate, the extent to which this advice would be translated into action may be questionable from previous literature. Walking is acceptable to patients[38] and it improves function [39], whilst it is also low impact, easily accessible,[39] adaptable to patient preferences and easy to incorporate into everyday life. However, it is unclear how acceptable or realistic it is for patients with CKP to engage in cycling or swimming.[40,41] For example, having to pay (e.g. for equipment or instructors) is a recognised barrier to physical activity engagement.[13,42] Combining the findings of uncertain appropriateness of (some) suggested modes of exercise with only 5% of GPs using general exercise explicitly stating that their advice should be tailored to their patients' interests and abilities, suggests a need for greater focus on individualising exercise. As many GPs reported having insufficient time and expertise to use exercise, referral to other HCPs may be appropriate. However physiotherapists report similar uncertainties about the safety and efficacy of exercise[19] and suboptimal use of both exercise types, favouring local more than general exercise approaches.[43] Further, it is unlikely to be an economically viable approach for GPs to manage all patients with CKP by referring them to physiotherapists. It is possible therefore, that solely relying on physiotherapists to deliver exercise interventions may not ensure all patients receive tailored, specific instruction on both exercise types. Finally, a third of respondents reported that they would opportunistically follow-up the vignette patient to check adherence to exercise. However, previous research examining the consultation behaviour of patients with CKP has shown that while many patients may consult again with other health problems, CKP is often not recorded again.[44] Thus the GPs' reported intentions regarding follow-up are likely to be unrealistically optimistic.

For peer review only - http://bmjopen.bmj.com/site/about/guidelines.xhtml

Strengths and limitations

This large UK survey is the first known to directly, specifically and concurrently investigate the attitudes, beliefs and behaviours of GPs regarding exercise for patients with CKP. The questionnaire was pre-tested and piloted before being used in this main study. Employing a vignette provided a consistent patient scenario to all GPs[45-48] and minimised the confounders inherent in observational research investigating clinical management using real patients.[46,47] Limitations of this study include the likely over-estimation of exercise use among GPs given the low response rate (response bias), the self-report nature of the questionnaire (social desirability bias) and the relatively uncomplicated vignette case. Due to the use of survey methodology, we could not explore the reasons underlying GPs responses. However, as GPs should be using exercise for all patients with CKP, the results of this survey are valuable for indicating an apparent evidence-practice gap in the way in which GPs employ exercise with this patient group.

Implications for future practice

Implementation of current evidence-based recommendations for the management of CKP needs to be improved among GPs in the UK. It is possible that at least part of the problem in implementing the recommendations is the lack of explicit guidance regarding the role of GPs, and other members of the multidisciplinary team who may be involved in, managing patients with this condition. This is perhaps reflected by the variable perceptions among GPs regarding their role in managing CKP and the extent to which they believe they should provide exercise advice or prescription. However, given the apparent association between perceived role and behaviour among the GPs who responded to the survey (reported elsewhere [17]), greater clarity of roles and expectations of all professional groups would be a good starting point for improving implementation of guidelines.

To deliver best practice for patients with CKP, strategies which target both GPs and the wider primary care team are needed. Two key areas should be addressed: i) development of

For peer review only - http://bmjopen.bmj.com/site/about/guidelines.xhtml

a pragmatic approach for GPs to initiate individualised local and general exercise and ii) identification of additional methods of initiating exercise and/or supporting patients to continue with exercise that do not solely rely on GPs. Given that theoretical patient behaviour change models[49] often involve a balance of perceived value and risks/burdens of undertaking the new behaviour, a pragmatic approach for GPs initiating exercise among patients with CKP would need to highlight the value of exercise to patients, its role relative to other interventions and practical ways to undertake specific and individualised exercise. Such a best practice approach was suggested by Khan et al[50] who recommended that GPs should encourage the use of exercise (e.g. by asking about physical activity at each consultation), consider the ‘5 A’s’ of physical activity counselling (assess, advise, agree, assist and arrange),[51] write an exercise prescription,[52-54] and refer or signpost to appropriate professionals or resources for exercise support and/or follow-up. Supplementary written leaflets or ‘guidebooks’ seem to be acceptable and useful[55] and can be accessed or signposted within consultations; for example, the Keele University OA guidebook[56] and the Arthritis Research UK knee OA booklet.[57] Given that CKP patients commonly have comorbidities[38,58,59] and multiple joint pain, GPs could use this opportunity to detect and manage comorbid conditions that may directly impact the use of exercise (e.g. cardiovascular disease, depression[60]), to relay the synergistic benefits of exercise for all relevant morbidities, and to make explicit that CKP should not prevent exercise for other conditions. Practical methods to help GPs provide the above information, in limited available time, need to be developed and may include personalised written care plans.[61] Given that time was the most frequently reported barrier to GPs initiating exercise, service delivery models may need to change such that exercise initiation, support and/or follow-up is primarily undertaken by other professionals such as physiotherapists,[62,63] practice nurses,[64] health trainers or local gym personnel. Direct access may enhance patients' utilisation of physiotherapists[65] and alternative, low GP burden, strategies could be explored to promote exercise use and/or follow-up. For example, technology enabled care

For peer review only - http://bmjopen.bmj.com/site/about/guidelines.xhtml

services[66] have shown promise when used to support exercise interventions among patients with cardiac[67] and chronic lung disease.[68]

CONCLUSIONS

Although the majority of UK GPs who responded to the questionnaire survey were positive about exercise for patients with CKP and used exercise in their clinical management, this survey identified GP uncertainties with respect to the safety and efficacy of exercise and suboptimal approaches to the initiation of exercise with patients. GPs' use of exercise may be improved by addressing the key barriers of time and expertise, by developing a pragmatic approach that supports GPs to initiate individualised exercise with patients, and/or by other professionals taking on this role.

ACKNOWLEDGEMENTS

This project was supported by Keele Clinical Trials Unit. We would also like to thank the staff within the Research Institute for Primary Care and Health Sciences who supported the study and the GPs who participated.

CONTRIBUTORSHIP STATEMENT

EC, NF, MP, TR, and ER participated in the design of the study, analysis of the results and helped to draft the manuscript. All authors read and approved the final manuscript.

COMPETING INTERESTS

The authors declare that they have no competing interests.

DATA SHARING

The datasets analysed during the current study available from the corresponding author on reasonable request.

FUNDING

For peer review only

For peer review only - http://bmjopen.bmj.com/site/about/guidelines.xhtml

This paper presents independent research funded by the Arthritis Research UK Centre in Primary Care grant (Grant Number 20202). NF, an NIHR Senior Investigator, is supported through an NIHR Research Professorship (NIHR-RP-011-015). EC was funded by an NIHR Academic Clinical Fellowship and subsequently by the NIHR School for Primary Care Research bridging funds. The views expressed in this publication are those of the authors and not necessarily those of the NHS, the NIHR or the Department of Health.

ETHICAL APPROVAL

Ethical approval for the study was obtained from Keele University (UK) Ethical Review Panel.

REFERENCES

(1) Jordan KM, Sawyer S, Coakley P, Smith HE, Cooper C, Arden NK. The use of conventional and complementary treatments for knee osteoarthritis in the community. *Rheumatology (Oxford)* 2004;43(3):381-4.

(2) Jinks C, Ong BN, Richardson J. A mixed methods study to investigate needs assessment for knee pain and disability: population and individual perspectives. *BMC Musculoskelet Disord* 2007;8:59.

(3) Thorstensson CA, Gooberman-Hill R, Adamson J, Williams S, Dieppe P. Help-seeking behaviour among people living with chronic hip or knee pain in the community. *BMC Musculoskelet Disord* 2009;10:153.

(4) National Institute for Health and Care Excellence. Osteoarthritis: care and management in adults. 2014;CG177.

(5) Cottrell E, Roddy E, Foster NE. The attitudes, beliefs and behaviours of GPs regarding exercise for chronic knee pain: a systematic review. *BMC Fam Pract* 2010;11(4).

(6) Vos T, Barber RM, Bell B, Bertozzi-Villa A, Biryukov S, Bolliger I, et al. Global, regional, and national incidence, prevalence, and years lived with disability for 301 acute and chronic diseases and injuries in 188 countries, 1990–2013: a systematic analysis for the Global Burden of Disease Study 2013. *Lancet* 2015 2016/03;386(9995):743-800.

(7) Jordan KP, Kadam UT, Hayward R, Porcheret M, Young C, Croft P. Annual consultation prevalence of regional musculoskeletal problems in primary care: an observational study. *BMC Musculoskelet Disord* 2010;11:144.

(8) Arthritis Research UK National Primary Care Centre. Musculoskeletal matters. Consultations for selected diagnoses and regional problems. Musculskeletal Matters 2010;Bulletin 2.

For peer review only - http://bmjopen.bmj.com/site/about/guidelines.xhtml

(9) McAlindon TE, Bannuru RR, Sullivan MC, Arden NK, Berenbaum F, Bierma-Zeinstra SM, et al. OARSI guidelines for the non-surgical management of knee osteoarthritis. *Osteoarthritis Cartilage* 2014;22(3):363-88.

(10) National Institute for Health and Care Excellence. Osteoarthritis NICE quality standard. QS87 ed. http://www.nice.org.uk/guidance/qs87: NICE; 2015.

(11) French SD, Bennell KL, Nicolson PJ, Hodges PW, Dobson FL, Hinman RS. What do people with knee or hip osteoarthritis need to know? An international consensus list of essential statements for osteoarthritis. *Arthritis Care Res (Hoboken)* 2015 May;67(6):809-16.

(12) Uthman OA, van der Windt DA, Jordan J, Dziedzic KS, Healey EL, Peat GM, et al. Exercise for lower limb osteoarthritis: systematic review incorporating trial sequential analysis and network meta-analysis. *BMJ* 2013;347:f5555.

(13) Mazieres B, Thevenon A, Coudeyre E, Chevalier X, Revel M, Rannou F. Adherence to, and results of, physical therapy programs in patients with hip or knee osteoarthritis. Development of French clinical practice guidelines. *Joint Bone Spine* 2008;75(5):589-96.

(14) Ajzen I. The theory of planned behavior. *Organ Behav Hum Decis Process* 1991 12;50(2):179-211.

(15) Michie S, Johnston M, Abraham C, Lawton R, Parker D, Walker A, et al. Making psychological theory useful for implementing evidence based practice: a consensus approach. *Qual Saf Health Care* 2005;14(1):26-33.

(16) Godin G, Bélanger-Gravel A, Eccles M, Grimshaw J. Healthcare professionals' intentions and behaviours: a systematic review of studies based on social cognitive theories. *Implement Sci* 2008;3:36.

(17) Cottrell E, Roddy E, Rathod T, Porcheret M, Foster NE. What influences general practitioners' use of exercise for patients with chronic knee pain? Results from a national survey. *BMC Fam Pract*. 2016;17:172.

(18) Cottrell E, Roddy E, Rathod T, Thomas E, Porcheret M, Foster NE. Maximising response from GPs to questionnaire surveys: do length or incentives make a difference? *BMC Med Res Methodol* 2015;15(1):3.

(19) Holden MA, Nicholls EE, Young J, Hay EM, Foster NE. UK-based physical therapists' attitudes and beliefs regarding exercise and knee osteoarthritis: findings from a mixed-methods study. *Arthritis Rheum* 2009;61(11):1511-21.

(20) Holden MA, Nicholls EE, Young J, Hay EM, Foster NE. Role of exercise for knee pain: what do older adults in the community think? *Arthritis Care Res (Hoboken)* 2012;64(10):1554-64.

(21) Roddy E, Zhang W, Doherty M, Arden NK, Barlow J, Birrell F, et al. Evidence-based recommendations for the role of exercise in the management of osteoarthritis of the hip or knee - the MOVE consensus. *Rheumatology (Oxford)* 2005;44(1):67-73.

(22) Zar J. Biostatistical analysis. 4th ed.: Prentice Hall; 1998.

For peer review only

For peer review only - http://bmjopen.bmj.com/site/about/guidelines.xhtml

(23) Northern Ireland Statistics & Research Agency. Northern Ireland Multiple Deprivation Measure 2010. 2010; Available at: http://www.nisra.gov.uk/deprivation/nimdm_2010.htm. Accessed January/06, 2015.

(24) Department for Communities and Local Government. English indices of deprivation 2010. 2011; . Accessed December/12, 2014.

(25) Welsh Government. Welsh Index of Multiple Deprivation (WIMD). 2014; Available at: http://wales.gov.uk/statistics-and-research/welsh-index-multiple-deprivation/?lang=en. Accessed January/06, 2015.

(26) The Scottish Government. Scottish Index of Multiple Deprivation. 2014; Available at: http://www.scotland.gov.uk/Topics/Statistics/SIMD. Accessed December/12, 2014.

(27) Mercer C, Jackson A, Moore A. Developing clinical guidelines for the physiotherapy management of whiplash associated disorder (WAD). *Int J Osteopath Med* 2007 2014/12;10(2):50-4.

(28) Fernandes L, Hagen KB, Bijlsma JWJ, Andreassen O, Christensen P, Conaghan PG, et al. EULAR recommendations for the non-pharmacological core management of hip and knee osteoarthritis. *Ann Rheum Dis* 2013;72(7):1125-35.

(29) Arshad A, Rashid R, Das Gupta E. A survey of the primary care management of osteoarthritis is Malaysia: a view from a rheumatologist's perspective. *Int J Rheum Dis* 2008;11(3):246-50.

(30) Richette P, Hilliquin P, Bertin P, Carni P, Berger V, Marty M. Comparison of general practitioners and rheumatologists' prescription patterns for patients wtih knee osteoarthritis. *BMC Musculoskelet Disord* 2011;12:72.

(31) Maserejian NN, Fischer MA, Trachtenberg FL, Yu J, Marceau LD, McKinlay JB, et al. Variations among primary care physicians in exercise advice, imaging, and analgesics for musculoskeletal pain: Results from a factorial experiment. *Arthritis Care Res (Hoboken)* 2014;66(1):147-56.

(32) Bedson J, Jordon K, Croft P. How do GPs use x rays to manage chronic knee pain in the elderly? A case study. *Ann Rheum Dis* 2003;62(5):450-4.

(33) Porcheret M, Jordan K, Croft P. Treatment of knee pain in older adults in primary care: development of an evidence-based model of care. *Rheumatology (Oxford)* 2007;46(4):638-48.

(34) Dexter PA. Joint exercises in elderly persons with symptomatic osteoarthritis of the hip or knee. Performance patterns, medical support patterns, and the relationship between exercising and medical care. *Arthritis Care Res* 1992;5(1):36-41.

(35) Mazzuca SA, Brandt KD, Katz BP, Dittus RS, Freund DA, Lubitz R, et al. Comparison of general internists, family physicians and rheumatologists managing patients with symptoms of osteoarthritis of the knee. *Arthritis Care Res* 1997;10(5):289-99.

(36) Glauser TA, Salinas GD, Roepke NL, Chad Williamson J, Reese A, Gutierrez G, et al. Management of mild-to-moderate osteoarthritis: a study of the primary care perspective. *Postgrad Med* 2011;123(1):126-34.

For peer review only - http://bmjopen.bmj.com/site/about/guidelines.xhtml

(37) Mamlin LA, Melfi CA, Parchman ML, Gutierrez B, Allen DI, Katz BP, et al. Management of osteoarthritis of the knee by primary care physicians. *Arch Fam Med* 1998;7(6):563-7.

(38) Holden MA, Nicholls EE, Young J, Hay EM, Foster NE. Exercise and physical activity in older adults with knee pain: a mixed methods study. *Rheumatology (Oxford)* 2015;54(3):413-23.

(39) O'Connor SR, Tully MA, Ryan B, Bleakley CM, Baxter GD, Bradley JM, et al. Walking exercise for chronic musculoskeletal pain: systematic eeview and meta-analysis. *Arch Phys Med Rehabil* 2015;96(4):724-34.e3.

(40) Hendry M, Williams NH, Markland D, Wilkinson C, Maddison P. Why should we exercise when our knees hurt? A qualitative study of primary care patients with osteoarthritis of the knee. *Fam Pract* 2006;23(5):558-67.

(41) Bossen D, Veenhof C, Dekker J, de Bakker D. The usability and preliminary effectiveness of a web-based physical activity intervention in patients with knee and/or hip osteoarthritis. *BMC Med Inform Decis Mak* 2013;13(1):1-8.

(42) Derananian CA, Desai P, Smith-Ray R, Seymour RB, Hughes SL. Perceived versus actual factors associated with adoption and maintenance of an evidence-based physical activity program. *Transl Behav Med* 2012;2(2):209-17.

(43) Holden MA, Nicholls EE, Hay EM, Foster NE. Physical therapists' use of therapeutic exercise for patients with clinical knee osteoarthritis in the United Kingdom: in line with current recommendations? *Phys Ther* 2008;88(10):1109-21.

(44) Bedson J, Mottram S, Thomas E, Peat G. Knee pain and osteoarthritis in the general population: what influences patients to consult? *Fam Pract* 2007;24(5):443-53.

(45) Peabody JW, Luck J, Glassman P, Dresselhaus TR, Lee M. Comparison of vignettes, standardized patients, and chart abstraction: a prospective validation study of 3 methods for measuring quality. *JAMA* 2000;283(13):1715-22.

(46) Norcini J. Back to the future: clinical vignettes and the measurement of physician performance. *Ann Intern Med* 2004;141(10):813-4.

(47) Veloski J, Tai S, Evans AS, Nash DB. Clinical vignette-based surveys: a tool for assessing physician practice variation. *Am J Med Qual* 2005;20(3):151-7.

(48) Peabody JW, Liu A. A cross-national comparison of the quality of clinical care using vignettes. *Health Policy Plan* 2007;22(5):294-302.

(49) Conner M, Normal P. Predicting and changing health behaviour: research and practice with social cognition models. 3rd Ed. ed. Maidenhead: Open University Press; 2015.

(50) Khan KM, Weiler R, Blair SN. Prescribing exercise in primary care: ten practical steps on how to do it. *BMJ* 2011;343:806-7.

(51) Peterson JA. Get moving! Physical activity counseling in primary care. *J Am Acad Nurse Pract* 2007;19(7):349-57.

For peer review only - http://bmjopen.bmj.com/site/about/guidelines.xhtml

(52) Grandes G, Sanchez A, Sanchez-Pinilla RO, Torcal J, Montoya I, Lizarraga K, et al. Effectiveness of physical activity advice and prescription by physicians in routine primary care: a cluster randomized trial. *Arch Intern Med* 2009;169(7):694-701.

(53) Professional Associations for Physical Activity (Sweden). Physical activity in the prevention and treatment of disease. Sweden: Swedish National Institute of Public Health; 2010.

(54) Patel A, Schofield GM, Kolt GS, Keogh JW. General practitioners' views and experiences of counselling for physical activity through the New Zealand Green Prescription program. *BMC Fam Pract* 2011;12:119.

(55) Morden A, Jinks C, Ong BN, Porcheret M, Dziedzic KS. Acceptability of a 'guidebook' for the management of Osteoarthritis: a qualitative study of patient and clinician's perspectives. *BMC Musculoskelet Disord* 2014;15:427.

(56) Keele University, Arthritis Research UK, National Institute for Health Research. A guide for people who have osteoarthritis. 2014; Available at: http://www.keele.ac.uk/media/keeleuniversity/ri/primarycare/pdfs/OA_Guidebook.pdf. Accessed July/03, 2015.

(57) Arthritis Research UK. Osteoarthritis of the knee. Chesterfield. Available at: http://www.arthritisresearchuk.org/arthritis-information/conditions/osteoarthritis-of-the-knee.aspx: Arthritis Research UK; 2013.

(58) Gore M, Tai KS, Sadosky A, Leslie D, Stacey BR. Use and costs of prescription medications and alternative treatments in patients with osteoarthritis and chronic low back pain in community-based settings. *Pain Pract* 2012;12(7):550-60.

(59) Brand CA, Harrison C, Tropea J, Hinman RS, Britt H, Bennell K. Management of osteoarthritis in general practice in Australia. *Arthritis Care Res (Hoboken)* 2014;66(4):551-8.

(60) Wilkie R, Blagojevic-Bucknall M, Jordan KP, Lacey R, McBeth J. Reasons why multimorbidity increases the risk of participation restriction in older adults with lower extremity osteoarthritis: a prospective cohort study in primary care. *Arthritis Care Res (Hoboken*) 2013;65(6):910-9.

(61) Goodwin N, Curry N, Naylor C, Ross S, Duldig W. Managing people with long-term conditions. The Kings Fund 2010:98.

(62) Hay EM, Foster NE, Thomas E, Peat G, Phelan M, Yates HE, et al. Effectiveness of community physiotherapy and enhanced pharmacy review for knee pain in people aged over 55 presenting to primary care: pragmatic randomised trial. *BMJ* 2006 Nov 11;333(7576):995.

(63) Hurley MV, Walsh NE, Mitchell HL, Pimm TJ, Patel A, Williamson E, et al. Clinical effectiveness of a rehabilitation program integrating exercise, self-management, and active coping strategies for chronic knee pain: a cluster randomized trial. *Arthritis Rheum* 2007;57(7):1211-9.

(64) Dziedzic KS, Healey EL, Porcheret M, Ong BN, Main CJ, Jordan KP, et al. Implementing the NICE osteoarthritis guidelines: a mixed methods study and cluster randomised trial of a model osteoarthritis consultation in primary care--the Management of OsteoArthritis In Consultations (MOSAICS) study protocol. *Implement Sci* 2014;9(1):95.

For peer review only - http://bmjopen.bmj.com/site/about/guidelines.xhtml

(65) Bury TJ, Stokes EK. Direct access and patient/client self-referral to physiotherapy: a review of contemporary practice within the European Union. *Physiotherapy* 2013;99(4):285-91.

(66) NHS England. Technology Enabled Care Services (TECS). Launch of technology enabled care services (TECS) resource for commissioners. Available at: https://www.england.nhs.uk/ourwork/qual-clin-lead/tecs/. Accessed April/05, 2016.

(67) Rawstorn JC, Gant N, Direito A, Beckmann C, Maddison R. Telehealth exercise-based cardiac rehabilitation: a systematic review and meta-analysis. *Heart* 2016 Mar 2.

(68) Hoaas H, Andreassen HK, Lien LA, Hjalmarsen A, Zanaboni P. Adherence and factors affecting satisfaction in long-term telerehabilitation for patients with chronic obstructive pulmonary disease: a mixed methods study. *BMC Med Inform Decis Mak* 2016 Feb 25;16(1):26-016-0264-9.

FIGURES

Figure 1 Flow-chart summarising the exercise types and initiation methods used by GPs' for the vignette patient with CKP

Figure 2 Barriers to using exercise reported by GPs

For peer review only

For peer review only - http://bmjopen.bmj.com/site/about/guidelines.xhtml

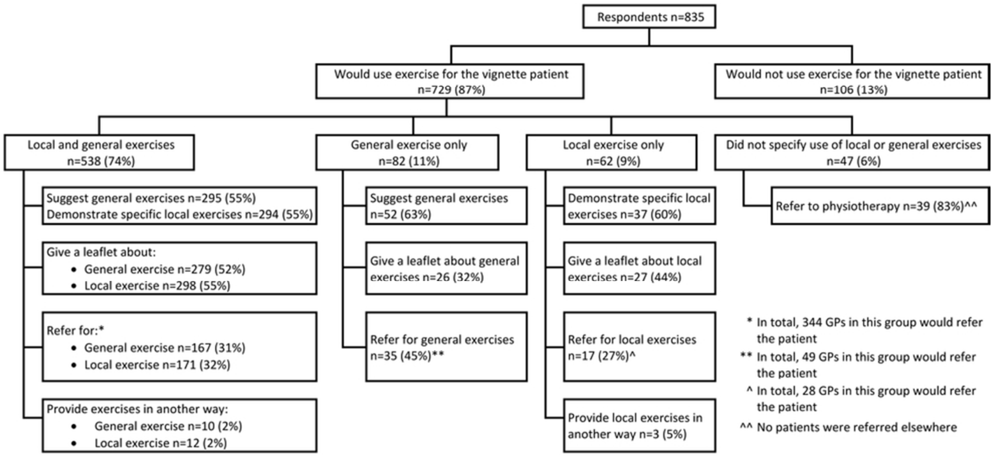

Figure 1 Flow-chart summarising the exercise types and initiation methods used by GPs' for the vignette patient with CKP

84x40mm (300 x 300 DPI)

For peer review only

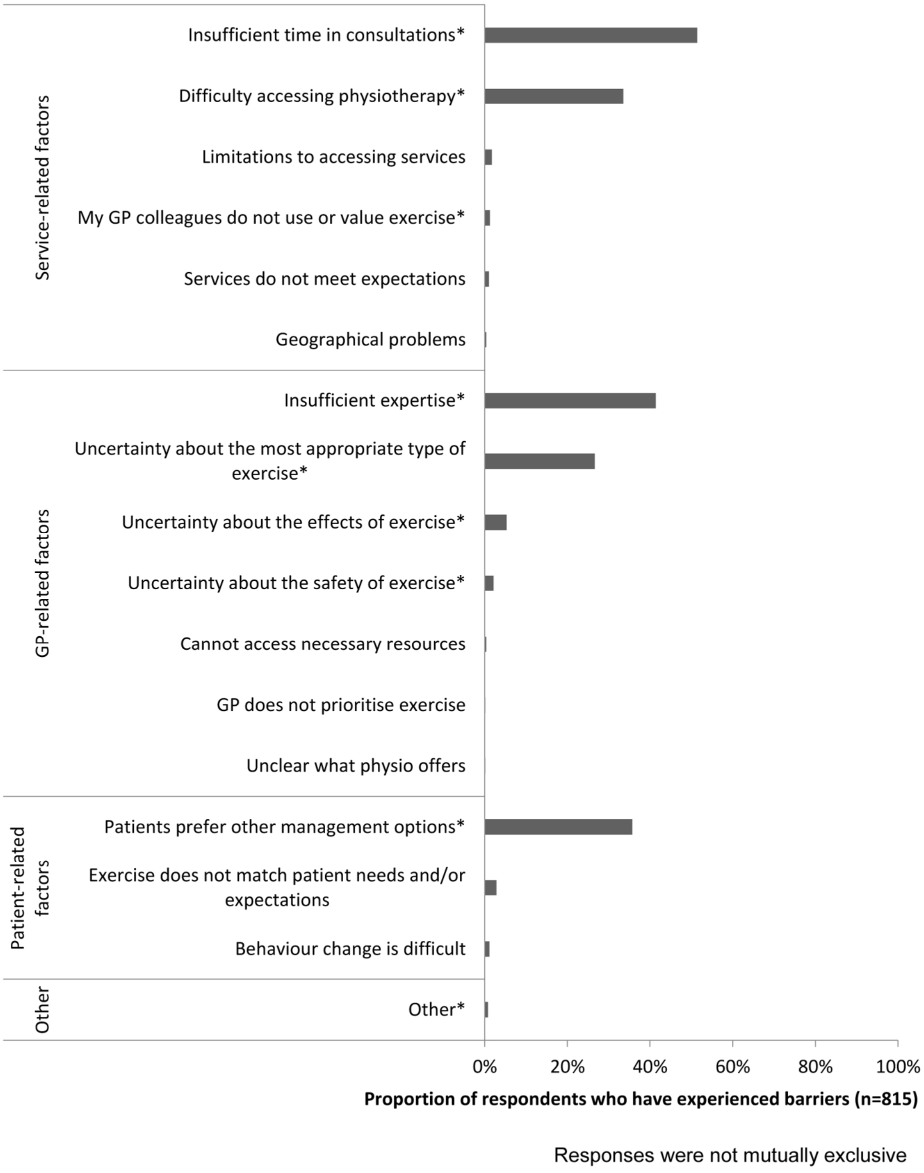

Figure 2 Barriers to using exercise reported by GPs
[asterisks denote barriers provided as a multiple response option in the questionnaire]

169x209mm (300 x 300 DPI)

Arthritis Research UK | primary care centre

Keele University

Management of Chronic Knee Pain Study

We are seeking the views of general practitioners who treat patients over 45 years old, with chronic knee pain.

If you **are not** a general practitioner, please tick this box and return the questionnaire without completing it any further ☐

If you **have not** managed someone with chronic knee pain in the last 6 months, please tick this box and return the questionnaire without completing it any further. ☐

This questionnaire should take no more than **20 minutes** to complete.

Return of your completed questionnaire will be interpreted as you providing your consent to participate in this study.

If you would like to participate in this study please either:

- Complete this paper version of the questionnaire and return it to Dr Elizabeth Cottrell, Academic Clinical Fellow GP Specialty Trainee, at the Arthritis Research UK Primary Care Centre, Keele University, Staffordshire, ST5 5BG using the enclosed FREEPOST envelope.
  OR
- Complete the online version of this questionnaire accessible at https://www.surveymonkey.com/s/Management_CKP

If you have any questions about this questionnaire or the study in general you can email Dr Elizabeth Cottrell at e.cottrell@keele.ac.uk

Instructions for completing this questionnaire

- When completing the questionnaire, please try and provide answers that most accurately reflect your usual clinical practice. There are no 'correct' or 'incorrect' answers.
- Where relevant please answer questions by ticking a box e.g. ☑
- Please do not consult any literature while completing this questionnaire.

Thank you for your help with this study

Unique survey ID	

For peer review only

For peer review only - http://bmjopen.bmj.com/site/about/guidelines.xhtml

Section 1: About you

1.1 Please state the year in which you qualified as a General Practitioner ☐

1.2 How many General Practitioners work in your practice (including yourself)? ☐

1.3 How do you best **describe yourself** (please tick **one box** only)

☐ GP Partner ☐ Salaried GP ☐ Locum GP

☐ Other, please specify ________

1.4 Is your practice ☐ Urban ☐ Semi-rural ☐ Rural

1.5 Are you ☐ Male ☐ Female

1.6 Are you a **GP with a special interest (GPwSI)** in musculoskeletal conditions?

☐ Yes ☐ No

1.7 Do you remember receiving any specific postgraduate musculoskeletal training which contained education about chronic knee pain? (By this we do not mean clinical placements or jobs in rheumatology or orthopaedics)

☐ Yes ☐ No

1.8 Do you have, or have you ever suffered from chronic knee pain **yourself**?

☐ Yes ☐ No

For peer review only

Section 2: Clinical scenario of a patient with chronic knee pain

Presented below is a clinical scenario of a patient **with chronic knee pain** who presents to you with this problem for the first time. All questions that follow relate to the care you would give this particular patient. Think about the patient's first consultation with you.

Patient: Mrs Jones, 58-year-old Prison Officer

History: First presentation of gradually worsening bilateral knee pain (right worse than left) over 2 years
No history of trauma
Pain always present when walking and at rest, worst when climbing stairs. No night pain.
Managing activities of daily living. Difficulty gardening.
Stopped going to gym – thinks was making pain worse
Only treatment tried is Ibuprofen once or twice when pain "really bad" – no benefit.
Came today finding work increasingly difficult due to the stairs
Usually well – no comorbidities

Medication: Nil

Examination: Body Mass Index 33
Knees – bilaterally no effusions. Joint tenderness upon palpation. Bilateral coarse crepitations.
Slightly reduced flexion of the right knee.
Hips – no abnormality detected

2.1 What **diagnosis** would you make at this point?

____

2.2 Using the words you would use with the patient, ***briefly*** state how you would **describe your diagnosis** to the patient

____

____

____

2.3 The patient's **symptoms** are: (please tick the **one** box that best reflects your opinion)

☐ Very severe ☐ Severe ☐ Moderate ☐ Mild ☐ Very mild

2.4 It is most likely that this patient's symptoms result from **knee damage** that is: (please tick the **one** box that best reflects your opinion)

☐ Very severe ☐ Severe ☐ Moderate ☐ Mild ☐ Very mild

2.5 Using the words you would use with the patient, ***briefly*** describe **what the future is likely to hold** with regards to her knee problem

____

____

2.6 What **investigation(s)/assessment(s)** would you do/order for this patient **at this point** and for what reason?

Please tick **all** you would undertake	Please indicate the reason for choosing this investigation (tick all that apply)					
	Reassure patient	To meet referral criteria	Confirm diagnosis	Inform treatment	Rule out other diagnoses	Inform prognosis
☐ None	---	---	---	---	---	---
☐ Blood tests	☐	☐	☐	☐	☐	☐
☐ Oxford knee score	☐	☐	☐	☐	☐	☐
☐ Knee x-ray	☐	☐	☐	☐	☐	☐
☐ Other - please state ______________	☐	☐	☐	☐	☐	☐

2.7 **At this consultation**, what approaches would you use, or suggest, to manage this patient? (please tick **all** that apply)

☐ Ice
☐ Keep active
☐ Weak opioids (e.g. codeine)
☐ Topical NSAID
☐ Heat
☐ Provision of walking stick(s)
☐ COX II inhibitor
☐ Paracetamol
☐ Acupuncture
☐ Advice on footwear
☐ Antidepressants
☐ Glucosamine/ chondroitin
☐ Rest
☐ Exercise
☐ Injection of steroids
☐ Topical capsaicin
☐ Weight loss
☐ Bed rest
☐ Oral non-selective NSAIDs (e.g. Ibuprofen)
☐ Strong opioids (e.g. morphine, Tramadol)
☐ None
☐ Transcutaneous electrical nerve stimulation

☐ Other (please state)

____

For peer review only

If you ticked "Exercise" in question 2.7 please go on to answer question 2.8. If not, please move straight on to question 2.9

2.8 There are a variety of approaches that can be used to manage chronic knee pain in general practice.

Of the following different approaches, **which, if any, would you use for this patient at this point**?

a) General exercises or increasing physical activity

☐ I would use ⇨ If you ***would use*** this strategy, please give details about what you would **actually do** (please tick any that apply)

- ☐ Suggest general exercises ⇨ Please state type of exercise ____________
- ☐ Give a leaflet
- ☐ Refer
- ☐ Other ⇨ Please state what other actions you would do ____________

☐ I would **not** use ⇨ If you ***would not use*** the strategy ***but would like to***, please tell us **what prevents** you (please tick any that apply)

- ☐ Insufficient expertise
- ☐ Insufficient time
- ☐ Other ⇨ Please state what other reasons prevent you (e.g. patient factors, access difficulties, uncertainty of benefit etc) ____________

b) Local knee or quadriceps strengthening exercises

☐ I would use ⇨ If you ***would use*** this strategy**,** please give details about what you would **actually do** (please tick any that apply)

- ☐ Demonstrate specific exercises
- ☐ Give a leaflet
- ☐ Refer
- ☐ Other ⇨ Please state what other actions you would do ____________

☐ I would **not** use ⇨ If you ***would not use*** the strategy ***but would like to***, please tell us **what prevents** you (please tick any that apply)

- ☐ Insufficient expertise
- ☐ Insufficient time
- ☐ Other ⇨ Please state what other reasons prevent you (e.g. patient factors, access difficulties, uncertainty of benefit etc) ____________

c) Follow up patient to check to see if she is undertaking exercise on a regular basis

☐ I would use ⇨ If you ***would use*** this strategy**,** please give details about what you would **actually do** (please tick any that apply)

- ☐ Planned follow-up ⇨
  - When: Please define number of days/weeks/months ____________
  - How: ☐ Face to face ☐ Via telephone
  - With whom: ☐ Yourself ☐ Another professional or service – please state ____________
- ☐ Opportunistic follow-up ⇨
  - When: ☐ If patient fails to improve ☐ Check when patient is next seen with this or any problem
- ☐ Other ⇨ Please state what other actions you would do ____________

☐ I would **not** use ⇨ If you ***would not use*** the strategy ***but would like to***, please tell us **what prevents** you (please tick any that apply)

- ☐ Insufficient expertise
- ☐ Insufficient time
- ☐ Other ⇨ Please state what other reasons prevent you ____________

For peer review only - http://bmjopen.bmj.com/site/about/guidelines.xhtml

2.9	Would you refer the patient to see someone else, either in the primary or community team or into secondary care, at this point?	☐ Yes	☐ No

If yes, to whom would you refer her?

____

2.10	Do you usually provide **written information** for patients in this situation?	☐ Yes	☐ No

If yes, please state the source of your written information

☐ Patient.co.uk or Emis Mentor ☐ Arthritis Research UK (previously known as ARC) or Arthritis Care ☐ Other, please state ____________

If you would provide written information, it would be very helpful to see a copy by returning it in the freepost envelope provided (**please write on your unique survey ID**) or by providing us with the link for any online information you access.

We are interested in your clinical opinion about patients **aged 45 years and over** with **chronic knee pain**. In this age group chronic knee pain is almost always due to knee osteoarthritis. Please answer all of the following questions using the definition of chronic knee pain as follows: knee pain and associated symptoms that have been present for more than 3 months not resulting from a fracture, infection, systemic rheumatological problem, metastases or surgery.

Section 3: Chronic knee pain in general practice

		I have never heard about or read it	I have heard of it but not seen it	I have seen it but not read it	I have read the full guideline and/or summary	I have read and consider the guideline when planning management
3.1	How much have you heard about or read the guideline published by NICE in 2008 for the care and management of osteoarthritis in adults? (please tick **one** box)	☐	☐	☐	☐	☐

Please consider **your role** in managing patients aged over 45 years old with chronic knee pain. Please indicate the extent to which you agree or disagree with the statements given by ticking **one** box per row.

		Strongly disagree	Disagree	Neither agree or disagree	Agree	Strongly agree
3.2	It is part of my job to manage people with chronic knee pain	☐	☐	☐	☐	☐
3.3	I have enough time to manage patients with chronic knee pain	☐	☐	☐	☐	☐
3.4	Managing patients with chronic knee pain is a priority for me	☐	☐	☐	☐	☐
3.5	Managing patients with chronic knee pain is of clinical interest to me	☐	☐	☐	☐	☐
3.6	It is part of my job to reassure patients about the safety of exercise for chronic knee pain	☐	☐	☐	☐	☐
3.7	It is part of my job to provide patients with chronic knee pain with a written management plan	☐	☐	☐	☐	☐

For peer review only - http://bmjopen.bmj.com/site/about/guidelines.xhtml

		I have no role in including exercise in the management	I inform patients that exercise is a management option	I advise patients to use exercise to manage their knee pain	I recommend the types of exercise patients could use	I give information on the type, frequency and duration of specific exercises
3.8	Which statement best describes your role in including exercise in the management plan of a patient with chronic knee pain? (please tick **one** box)	☐	☐	☐	☐	☐

3.9 We are interested to hear about **your experiences of barriers which might prevent the use of exercise** in your management of chronic knee pain. Please tick **all** of the barriers that you have experienced

☐ Insufficient time in consultations
☐ Insufficient expertise to give detailed information
☐ Difficulty accessing physiotherapy
☐ Uncertainty about the effects of exercise
☐ Patients prefer other management options
☐ My GP colleagues do not use or value exercise
☐ Uncertainty about the most appropriate type of exercise
☐ Uncertainty about the safety of exercise
☐ Other (please state) ____________________

____

Section 4: Your views about chronic knee pain

Below is a list of **possible causes** for a patient developing chronic knee pain. Please indicate the extent to which you agree or disagree with these causes by ticking **one** box in each row.

		Strongly disagree	Disagree	Neither agree or disagree	Agree	Strongly agree
4.1	Hereditary/runs in the family	☐	☐	☐	☐	☐
4.2	Being overweight/obese	☐	☐	☐	☐	☐
4.3	A person's own mental attitude e.g. thinking about life negatively	☐	☐	☐	☐	☐
4.4	A person's emotional state e.g. feeling down, anxious	☐	☐	☐	☐	☐
4.5	Ageing	☐	☐	☐	☐	☐
4.6	Accident or injury	☐	☐	☐	☐	☐
4.7	Manual work	☐	☐	☐	☐	☐
4.8	Sport	☐	☐	☐	☐	☐
4.9	Osteoarthritis	☐	☐	☐	☐	☐
4.10	Changes consistent with osteoarthritis seen on x-ray	☐	☐	☐	☐	☐

For peer review only

When completing the following questions, please consider patients **aged over 45 years old** with **chronic knee pain**. Please indicate the extent to which you agree or disagree with the statements given by ticking one box per row.

		Totally disagree	Largely disagree	Disagree to some extent	Agree to some extent	Largely agree	Totally agree
4.11	Mental stress can cause chronic knee pain even in the absence of tissue damage	☐	☐	☐	☐	☐	☐
4.12	The cause of chronic knee pain is unknown	☐	☐	☐	☐	☐	☐
4.13	Pain is a nociceptive stimulus, indicating tissue damage	☐	☐	☐	☐	☐	☐
4.14	A patient suffering from severe chronic knee pain will benefit from physical exercise	☐	☐	☐	☐	☐	☐
4.15	Functional limitations associated with chronic knee pain are the result of psychosocial factors	☐	☐	☐	☐	☐	☐
4.16	Patients with chronic knee pain should preferably practice only pain free movements	☐	☐	☐	☐	☐	☐
4.17	Therapy may have been successful even if pain remains	☐	☐	☐	☐	☐	☐
4.18	Chronic knee pain indicates the presence of organic injury	☐	☐	☐	☐	☐	☐
4.19	If chronic knee pain increases in severity, I immediately adjust the intensity of my treatment accordingly	☐	☐	☐	☐	☐	☐
4.20	If therapy does not result in a reduction in chronic knee pain, there is a high risk of severe restrictions in the long term	☐	☐	☐	☐	☐	☐
4.21	Pain reduction is a precondition for the restoration of normal functioning	☐	☐	☐	☐	☐	☐
4.22	Increased pain indicates new tissue damage or the spread of existing damage	☐	☐	☐	☐	☐	☐
4.23	There is no effective treatment to eliminate chronic knee pain	☐	☐	☐	☐	☐	☐
4.24	Even if the pain has worsened, the intensity of the next treatment can be increased	☐	☐	☐	☐	☐	☐
4.25	If patients complain of pain during exercise, I worry that damage is being caused	☐	☐	☐	☐	☐	☐
4.26	The severity of tissue damage determines the level of pain	☐	☐	☐	☐	☐	☐
4.27	Learning to cope with stress promotes recovery from chronic knee pain	☐	☐	☐	☐	☐	☐
4.28	Exercises that may be knee straining should not be avoided	☐	☐	☐	☐	☐	☐
4.29	In the long run, patients with chronic knee pain have a higher risk of developing severe functional impairments	☐	☐	☐	☐	☐	☐

For peer review only - http://bmjopen.bmj.com/site/about/guidelines.xhtml

Section 5: Your views about the role of exercise in treating chronic knee pain

We are interested in your views about the **role of exercise** in the treatment of **chronic knee pain in patients over 45 years old**. Please indicate the extent to which you agree or disagree with the statements given by ticking one box per row.

		Strongly disagree	Disagree	Neither agree or disagree	Agree	Strongly agree
5.1	GPs should prescribe quadriceps strengthening exercises to every patient with chronic knee pain	☐	☐	☐	☐	☐
5.2	GPs should prescribe general exercise, for example, walking or swimming, for every patient with chronic knee pain	☐	☐	☐	☐	☐
5.3	Knee problems are improved by quadriceps strengthening exercises	☐	☐	☐	☐	☐
5.4	Knee problems are improved by general exercise, for example walking or swimming	☐	☐	☐	☐	☐
5.5	Quadriceps strengthening exercises for the knee are safe for everybody to do	☐	☐	☐	☐	☐
5.6	General exercise, for example walking or swimming is safe for everybody to do	☐	☐	☐	☐	☐
5.7	Exercise for chronic knee pain is most beneficial when it is tailored to meet individual patient needs	☐	☐	☐	☐	☐
5.8	A standard set of exercises is sufficient for every patient with chronic knee problems	☐	☐	☐	☐	☐
5.9	GPs should educate chronic knee pain patients about how to change their lifestyle for the better	☐	☐	☐	☐	☐
5.10	It is important that people with chronic knee pain increase their overall activity levels	☐	☐	☐	☐	☐
5.11	How well a patient complies with their exercise programme determines how effective it will be	☐	☐	☐	☐	☐
5.12	GPs should follow up patients to monitor extent of continuation of exercises	☐	☐	☐	☐	☐
5.13	It is the patient’s own responsibility to continue doing their exercise programme	☐	☐	☐	☐	☐
5.14	Exercise is effective for patients if an x-ray shows severe knee osteoarthritis	☐	☐	☐	☐	☐
5.15	Exercise works just as well for everybody, regardless of the amount of pain they have	☐	☐	☐	☐	☐
5.16	Increasing the strength of the muscles around the knee stops the knee problem getting worse	☐	☐	☐	☐	☐
5.17	Increasing overall activity levels stops the knee problem getting worse	☐	☐	☐	☐	☐
5.18	Exercise for chronic knee pain is more effectively provided by physiotherapists than GPs	☐	☐	☐	☐	☐
5.19	Time constraints prevent GPs from providing advice on individual exercises for chronic knee pain	☐	☐	☐	☐	☐
5.20	Exercise for chronic knee pain should preferably be used after drug treatment has been tried	☐	☐	☐	☐	☐
5.21	Exercise for chronic knee pain would be used more frequently if access to physiotherapy was easier	☐	☐	☐	☐	☐

For peer review only - http://bmjopen.bmj.com/site/about/guidelines.xhtml

Would you be happy for us to contact you again in the future regarding this study?

☐ Yes ☐ No

If you answered YES to the question above please provide your name and contact details below (these details will be kept separately from your responses to the questionnaire):

**Name:**	
**Daytime telephone number:**	
**Address:**	
**Email:**	

End of Questionnaire

You have reached the end of the questionnaire. Please return the questionnaire in the **FREEPOST** envelope provided.

If you have any questions about this questionnaire or the study in general, you can email Dr Elizabeth Cottrell at e.cottrell@keele.ac.uk

Thank you for taking the time to complete this questionnaire. Your time and participation is greatly appreciated.

Unique survey ID

For peer review only
